# Challenges in estimating HIV prevalence trends and geographical variation in HIV prevalence using antenatal data: Insights from mathematical modelling

**DOI:** 10.1371/journal.pone.0242595

**Published:** 2020-11-20

**Authors:** Leigh F. Johnson, Mmamapudi Kubjane, Jeffrey W. Eaton

**Affiliations:** 1 Centre for Infectious Disease Epidemiology and Research, University of Cape Town, Cape Town, South Africa; 2 MRC Centre for Global Infectious Disease Analysis, Department of Infectious Disease Epidemiology, Imperial College London, London, United Kingdom; British Columbia Centre for Excellence in HIV/AIDS, CANADA

## Abstract

**Background:**

HIV prevalence data among pregnant women have been critical to estimating HIV trends and geographical patterns of HIV in many African countries. Although antenatal HIV prevalence data are known to be biased representations of HIV prevalence in the general population, mathematical models have made various adjustments to control for known sources of bias, including the effect of HIV on fertility, the age profile of pregnant women and sexual experience.

**Methods and findings:**

We assessed whether assumptions about antenatal bias affect conclusions about trends and geographical variation in HIV prevalence, using simulated datasets generated by an agent-based model of HIV and fertility in South Africa. Results suggest that even when controlling for age and other previously-considered sources of bias, antenatal bias in South Africa has not been constant over time, and trends in bias differ substantially by age. Differences in the average duration of infection explain much of this variation. We propose an HIV duration-adjusted measure of antenatal bias that is more stable, which yields higher estimates of HIV incidence in recent years and at older ages. Simpler measures of antenatal bias, which are not age-adjusted, yield estimates of HIV prevalence and incidence that are too high in the early stages of the HIV epidemic, and that are less precise. Antenatal bias in South Africa is substantially greater in urban areas than in rural areas.

**Conclusions:**

Age-standardized approaches to defining antenatal bias are likely to improve precision in model-based estimates, and further recency adjustments increase estimates of HIV incidence in recent years and at older ages. Incompletely adjusting for changing antenatal bias may explain why previous model estimates overstated the early HIV burden in South Africa. New assays to estimate the fraction of HIV-positive pregnant women who are recently infected could play an important role in better estimating antenatal bias.

## Introduction

A major goal of the Joint United Nations Programme on HIV/AIDS (UNAIDS) is to reduce the annual number of new HIV infections by 75% over the 2010–2020 period, and by 90% over the 2010–2030 period [1, 2]. However, monitoring of progress towards this and other HIV incidence targets is challenging. Antenatal HIV prevalence data have been critical to the estimation of trends in HIV prevalence and incidence in countries with generalized HIV epidemics [3–6], but because these data are representative only of pregnant women, they are known to be biased representations of HIV prevalence patterns and trends. Since the early 2000s, many countries in sub-Saharan Africa have conducted national household-based surveys, which are considered to be the ‘gold standard’ in evaluating HIV prevalence patterns and trends [7, 8]. However, the infrequency of these household surveys and the absence of household survey data from the period before 2000 means that antenatal HIV prevalence data remain very important in estimating prevalence and incidence trends. In recent years, many countries have switched from conducting HIV testing in samples of pregnant women to reporting programme statistics on the proportion of all women testing positive as part of prevention of mother-to-child transmission (PMTCT) programmes, thus increasing the availability of antenatal HIV prevalence data [5, 9].

Mathematical models are frequently fitted to antenatal HIV prevalence data in order to produce estimates of HIV prevalence and incidence trends. However, very few of these models simulate the relationship between HIV and pregnancy in any detail. Instead, models have attempted to account for the difference in HIV prevalence between pregnant women and the general population (‘antenatal bias’) in a number of ways. Several models [10–12] have controlled for the fact that HIV reduces fertility (particularly in the more advanced stages of HIV infection [13–15]), which implies that antenatal HIV prevalence data may understate true HIV prevalence levels in women; some of these models [10, 12] also make provision for a restoration of fertility after women start ART, and hence a reduction in the extent of the understatement as more women receive ART. Some models [10, 11] also control for the fact that antenatal surveys represent only sexually experienced women, and thus may overstate HIV prevalence (particularly in younger age groups, where a substantial fraction of women may be virgins). In addition, a few models are fitted to age-specific antenatal HIV prevalence data rather than aggregated antenatal HIV data [10, 11], recognizing that HIV prevalence trends may differ by age, and that antenatal HIV data are mainly representative of younger women [16]. However, few models have considered other potential sources of bias, and there has been little effort to model antenatal bias mechanistically, using models that realistically simulate both HIV acquisition and fertility. A particular concern is that when a high proportion of HIV infections have been recently acquired (for example in early-stage HIV epidemics and in younger women), one might expect a strong positive correlation between HIV and fertility, as both reflect recent unprotected sex. Another concern is that changes in sexual behaviour could change both HIV incidence and fertility, potentially changing the extent of antenatal bias [17]. Another significant determinant of fertility is the duration of breastfeeding [18], which has reduced substantially in HIV-positive women as a result of PMTCT programmes [19, 20]. Across sub-Saharan Africa there have been substantial increases in modern contraceptive use since 1990 [21], and these could also potentially change patterns of antenatal bias. If models do not control for these sources of bias, there is the risk that they may incorrectly estimate HIV prevalence and incidence trends.

Antenatal HIV prevalence data are also used to make inferences about differences in HIV prevalence across countries and within countries [22–24], which are critical in deciding how to allocate resources across regions [25]. However, previous studies have shown that the extent of the antenatal bias may differ both across countries [8, 26] and within countries [26, 27]. For example, Marsh *et al*. [26] found that antenatal bias was generally higher in rural areas than in urban areas (though not in Southern African countries), and that antenatal bias was generally greatest in Western and Central Africa. It is important to understand the factors that account for antenatal bias in order to appropriately adjust for likely differences in the extent of antenatal bias across settings.

This study aims to understand antenatal bias in South Africa, the country with the largest number of HIV infections in the world [28], and a country that continues to rely heavily on antenatal HIV prevalence data for the purpose of producing HIV estimates [27]. Firstly, we aim to compare different definitions of antenatal bias, and to assess whether antenatal bias changes over time. Secondly, we aim to evaluate the extent to which different factors drive antenatal bias, and how these factors influence changes in antenatal bias over time. Thirdly, we assess age differences in antenatal bias, and propose an alternative definition of antenatal bias that is more stable across age groups and over time. Fourthly, we aim to assess how antenatal bias differs between urban and rural South African communities. Finally, we aim to assess how model estimates of HIV prevalence and incidence trends change depending on the assumptions made about antenatal bias in the calibration process.

## Materials and methods

Our approach is to use simulated datasets, generated by an agent-based model of HIV and fertility in South Africa (MicroCOSM), to characterize the factors that contribute to antenatal bias. We then test the effect of different assumptions about antenatal bias using a deterministic model, Thembisa, which is typical of models used to estimate HIV prevalence trends from antenatal HIV data. Whereas MicroCOSM simulates fertility (and its association with HIV) in much detail, Thembisa relies on simple assumptions about age-specific fertility rates and effects of HIV on fertility, without directly simulating the common determinants of fertility and HIV transmission. Assumptions about the nature of antenatal bias are therefore required (as inputs) in the Thembisa model, but not in MicroCOSM. Antenatal bias, defined in different ways, can instead be calculated as an output of MicroCOSM. Although Thembisa requires assumptions about the nature of antenatal bias, it does not depend directly on the MicroCOSM outputs.

### The MicroCOSM model

MicroCOSM (Microsimulation for the Control of South African Morbidity and Mortality) simulates a nationally-representative sample of the South African population, starting in 1985 [29]. The initial sample consists of 20 000 individuals, and the simulated population size changes as births and deaths occur. A number of the HIV, contraception and sexual behaviour parameters are assumed to change over time (as summarized in Table 1). Each sampled individual in the population is assigned a number of demographic variables (date of birth, sex and race), as well as socio-economic variables that can change over time. Each individual is also assigned a number of sexual behaviour characteristics, some of which are assumed fixed (e.g. risk group, which is defined in terms of propensity for concurrent partnerships and commercial sex), and some of which vary over the course of the simulation (e.g. number of current partners, sexual preference, marital status and consistency of condom use with current partners). At weekly time steps the sexual activity of the simulated population is updated, allowing for termination of existing partnerships, formation of new partnerships, changes in relationship type (from short-term to married) and once-off sexual contacts. A more detailed description of the sexual behaviour model is provided elsewhere [29].

**Table 1:** Time-dependent variables in the MicroCOSM model.

Parameter	Values	Data sources
% of girls aged 15–19 using condoms consistently with new short-term partners	1% (1985) to 42% (2007) to 30% (2017)	Calibration to national survey data (condom use at last sex/for contraception), adjusting for mis-reporting [29]
% of pregnant women offered testing for HIV	0% (2000) to 90% (2005+)	District Health Information System [30]
% of HIV-diagnosed mothers who breastfeed	44% (2000) to 80% (2013+)	National PMTCT surveys [31, 32]
% of ART-eligible patients who start ART soon after diagnosis (asymptomatic)*	1% (2000) to 40% (2012+)	Rates of ART initiation soon after diagnosis in a systematic review of SA studies [33]
Relative rate of female sterilization (relative to 1997)	(1997) to 0.5 (2007+)	DHS data [34–36]
Odds of adopting hormonal contraception among women aged 15–24 (relative to 1997)	1.0 (1997) to 0.2 (2005+)	Calibration to DHS [34–36] and other survey data [37, 38]
Specificity of HIV testing algorithm in ANC surveys	100% (1996) to 97.7% (1997+)	Bayesian analysis of ANC survey data [39]

* Higher rates apply in symptomatic patients and pregnant women; individuals can also link to ART at later durations after diagnosis. ANC = antenatal clinic. ART = antiretroviral treatment. DHS = demographic and health survey. PMTCT = prevention of mother-to-child transmission.

HIV is introduced into the simulated population in 1990, the same year that the first antenatal survey was conducted in South Africa. A small initial fraction of the high-risk population (approximately 2%) is assigned an HIV-positive status, and each infected individual is randomly assigned an initial HIV viral load and CD4 count, which are subsequently updated at weekly time steps. HIV transmission is modelled based on an assumed transmission probability per unprotected sex act, which depends on the HIV-positive partner’s viral load, sex and type of sex act, male circumcision (if the susceptible partner is a male engaging in heterosexual intercourse), age (if the susceptible partner is female) and the presence of other sexually transmitted infections (STIs), which are dynamically simulated in the same way as HIV. In addition, the model assumes that pregnant women have an HIV acquisition risk 1.75 times that in non-pregnant women [40–42], and that women who use injectable contraceptives have an acquisition risk 1.28 times that in women who are using other non-barrier contraception or no contraception [43]. After HIV acquisition, HIV-related mortality probabilities are calculated at weekly time steps, with untreated mortality rates being assumed to depend on the individual’s current CD4 count.

The model allows for a number of HIV interventions. HIV communication programmes, condom distribution and life skills programmes in schools are assumed to have led to increased levels of condom use since the mid-1990s [10], though the model also makes provision for some decline in condom use in recent years (Table 1). HIV testing is assumed to have increased steadily since 1990. HIV-diagnosed individuals are assumed to be more likely to use condoms consistently, depending on whether they disclose their HIV status to their sexual partner(s), and HIV-diagnosed mothers are assumed to be less likely to breastfeed than HIV-negative mothers. PMTCT programmes are assumed to have led to increased HIV diagnosis among HIV-positive mothers, as well as reduced mother-to-child transmission, in part because of increased antiretroviral prophylaxis and in part because of reduced breastfeeding. However, the withdrawal of free formula milk from public antenatal clinics since 2011 is assumed to have contributed to a subsequent increase in breastfeeding by HIV-positive mothers [32]. Antiretroviral treatment (ART) is assumed to have been introduced in the public health sector since 2004, with associated reductions in both HIV mortality and HIV transmission risk.

In addition to condom use, the model simulates the use of three different types of non-barrier contraception: oral contraception, injectable contraception and female sterilization. Rates of sterilization in sexually active women are assumed to have dropped by 50% over the 1997–2007 period, in order to match an observed decline in the fraction of women who are sterilized [36]. Most hormonal contraceptive initiation is assumed to coincide with either (a) the formation of a new partnership, or (b) delivery or weaning. In the case of (a), the model assumes that women are less likely to adopt hormonal contraception if they are using condoms consistently, but if the couple subsequently discontinues consistent condom use the model allows for possible initiation of hormonal contraception to ‘replace’ condoms. Discontinuation of hormonal contraception is assumed to occur in the absence of recent sexual activity, on falling pregnant, on getting sterilized, or for other reasons (mostly desire for children or dissatisfaction with current contraception). The model has been calibrated to patterns of contraceptive use reported in Demographic and Health Surveys (DHSs) [34–36, 44] and other South African surveys [37, 38]. In order to match the model to the observed trends, it was necessary to assume an 80% reduction in the uptake of hormonal contraception by young women between 1997 and 2005.

A woman’s risk of falling pregnant is assumed to be proportional to her number of current partners and frequency of unprotected contact with those partners. The risk of falling pregnant is assumed to be reduced by 78% if the woman uses condoms or oral contraceptives, by 90% if the woman uses injectable contraceptives and by 99.5% if the woman is sterilized [45–49]. The incidence of pregnancy is also scaled by an individual-specific fecundability parameter, which varies with respect to age (increasing as girls reach menarche and decreasing as women reach menopause) and between women (allowing for natural variability in fertility [50]). Fertility rates in untreated HIV-positive mothers are assumed to be reduced 8%, 20% and 27% at CD4 counts of ≥350, 200–349 and <200 cells/μl respectively, but women on ART are assumed to have a 20% higher fertility rate than HIV-negative women [13]. Women are assumed not to be at risk of falling pregnant if they are currently breastfeeding. Rates of conception are scaled in such a way that the model matches previously-estimated rates of fertility in South Africa, by age, race and calendar period [51].

**Fig 1:**
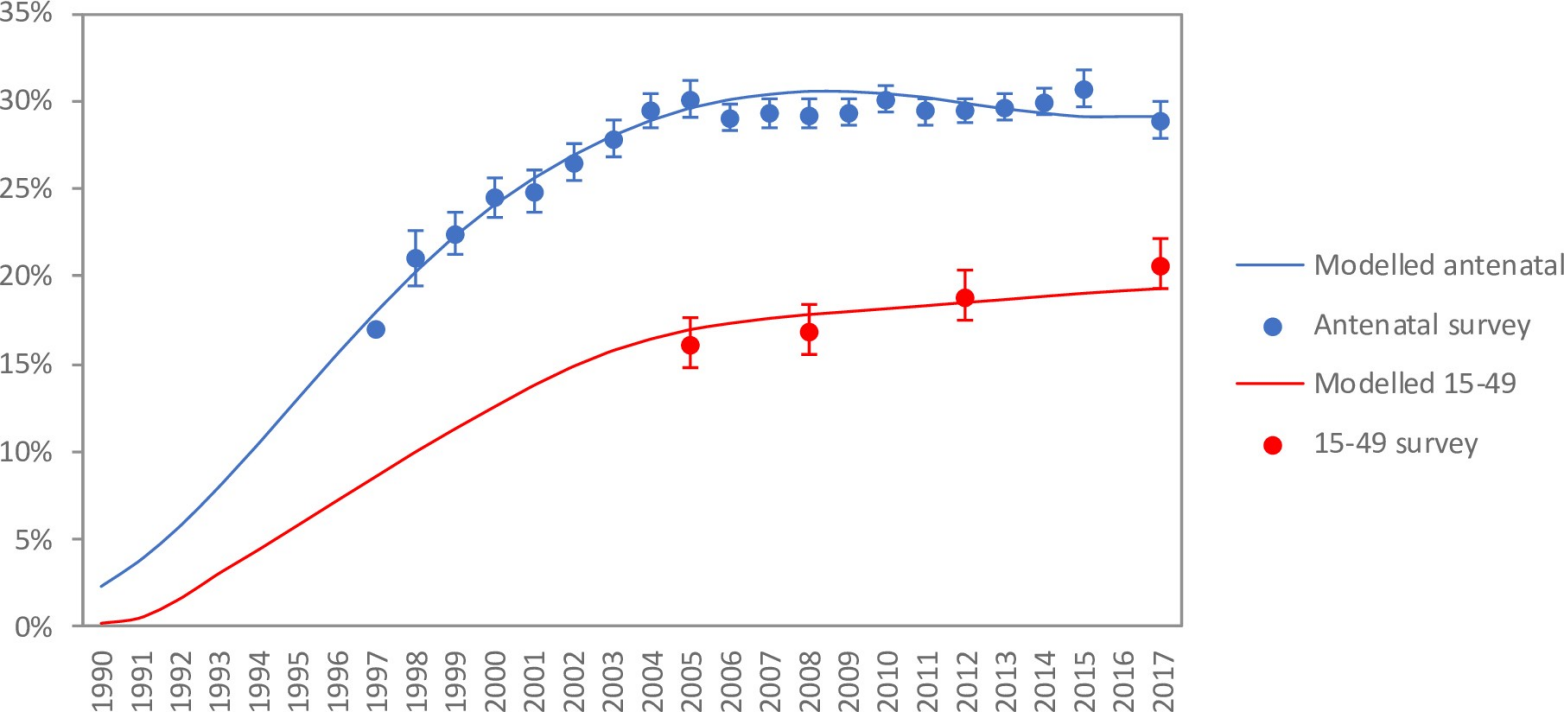
Comparison of HIV prevalence in pregnant women and population aged 15–49.

The model has been fitted to age-specific antenatal HIV prevalence data from the 1997–2015 period, as well as age- and sex-specific HIV prevalence data from national household surveys conducted in 2005, 2008 and 2012. Although antenatal HIV survey data are also available for the pre-1997 period, these early surveys have not been included because they did not follow a standard sampling protocol, and are probably biased towards urban antenatal clinics. Although the early surveys included retesting to confirm positive results, surveys conducted since 1997 have relied on a single test per woman, with no confirmatory testing. In calibrating the model to the antenatal data, we have therefore adjusted the model estimates of HIV prevalence in pregnant women to allow for an assumed 97.7% specificity [39]. We have also adjusted the model estimates of HIV prevalence in pregnant women to take account of racial differences in the proportion of pregnant women who use public antenatal clinics: 86.2% in African women, 77.1% in women of mixed race and 11.0% in white and Asian women (based on 1998 and 2016 DHS data). The model was calibrated by specifying prior distributions (representing ranges of uncertainty) for 12 different HIV transmission and behaviour parameters, and sampling 48 000 parameter combinations from these parameter combinations. For each parameter combination, the model was run, and a likelihood statistic was calculated to represent the level of consistency between the model estimates of HIV prevalence in pregnant women (after adjustment for specificity and public sector bias) and the antenatal survey estimates, as well as the consistency between the model estimates and data from other HIV surveys. The 100 parameter combinations that yielded the highest likelihood values were selected, and in the sections that follow, the results presented are the average results calculated from these 100 parameter combinations. A more detailed description of the model and calibration procedure is provided elsewhere [29]. Fig 1 and S1 and S2 Figs in S1 File show the model fits to the antenatal and household survey HIV prevalence data.

### Definitions of antenatal bias

We consider three definitions of antenatal bias. The first definition (‘unadjusted definition’) is the definition of antenatal bias used in a number of UNAIDS Reference Group publications [4, 8, 26]: the difference between HIV prevalence in pregnant women and adults aged 15–49, on a probit scale. Mathematically, if *ρ*_*t*_ is the HIV prevalence in the population aged 15–49 in year *t*, and *α*_*t*_ is the HIV prevalence in pregnant women in year *t*, the antenatal bias in year *t* is

$$c_{1}(t)=\Phi^{-1}(\alpha_{t})-\Phi^{-1}(\rho_{t}),$$

where Φ^-1^(*x*) represents the probit transformation of *x*. This measure does not adjust for any of the factors that might account for changes in antenatal bias over time (age differences in samples, effects of HIV on fertility, differences in sexual behaviour, etc.). Note that *α*_*t*_ is the MicroCOSM model simulation of the HIV prevalence in a survey of pregnant women attending public antenatal clinics, not the actual HIV prevalence in previously-published survey reports. In sensitivity analysis, we consider the effect of calculating the difference in prevalence on a natural log scale rather than on a probit scale (i.e. ln(*α*_*t*_)–ln(*ρ*_*t*_)).

The second definition (‘adjusted definition’) adjusts for a number of the factors that are known confounders, including age differences between pregnant women and women in the general population, the effect of HIV on fertility, and differences in sexual experience between pregnant women and women in the general population. This definition is more applicable to recent models that attempt to control for these sources of bias [10, 12]. Suppose that *γ*_*s*_(*x*,*t*) represents the fraction of sexually experienced women aged *x* to *x*+4 in year *t*, who are HIV-positive and in stage *s* of HIV infection. If *ρ'*(*x*,*t*) is the expected HIV prevalence in pregnant women aged *x* to *x*+4 in year *t*, then

$$\rho^{\prime }(x,t)=\frac{\sum_{s}\gamma_{s}(x,t)R(s)}{1+\sum_{s}\gamma_{s}(x,t)\left(R(s)-1\right)},$$

where *R*(*s*) is the ratio of HIV-positive fertility in HIV stage *s* to HIV-negative fertility in women of the same age. The *ρ'*(*x*,*t*) variable therefore represents the HIV prevalence that would be expected in pregnant women if sexual experience (implicit in the *γ*_*s*_(*x*,*t*) term) and HIV stage were the only factors affecting women’s fertility. For the sake of simplicity, we assume values of *R*(*s*) that are the same as in MicroCOSM (i.e. values of 0.92, 0.80 and 0.73 for untreated CD4 counts of ≥350, 200–349 and <200 cells/μl respectively, and 1.20 for women on ART). Then if *α*(*x*,*t*) is the antenatal prevalence measured in year *t* in women aged *x* to *x*+4, the antenatal bias in year *t* and in age group *x* to *x*+4 is

$$c_{2}(x,t)=\Phi^{-1}(\alpha (x,t))-\Phi^{-1}(\rho^{\prime }(x,t)),$$

and the average antenatal bias in year *t* is

$$c_{2}(t)=\frac{1}{5}\sum_{x}c_{2}(x,t),$$

where the summation is across the 5 age groups 15–19, 20–24, 25–29, 30–34 and 35–39 (we do not include women over the age of 40 as these are typically a small fraction of all pregnant women). Again note that *α*(*x*,*t*) is the model simulation of the prevalence that would be measured in a sample of pregnant women attending public antenatal clinics, not the actual prevalence measured in the antenatal surveys. As before, a sensitivity analysis is conducted to assess the effect of defining the antenatal bias on the natural log scale instead of the probit scale.

The third definition (‘recency-adjusted definition’) is similar to the second but aims to adjust also for potential bias due to the recency of infection (discussed below). If ρ*(*x*,*t*) is the recency-adjusted expectation of HIV prevalence in pregnant women aged *x* to *x*+4 in year *t*, this is calculated as ρ*(*x*,*t*) = *ρ'*(*x*,*t*) × *λ*(*x*,*t*)^*θ*^, where *λ*(*x*,*t*) is the fraction of sexually experienced HIV-positive women aged *x* to *x*+4 who were infected in the last 12 months, at time *t*, and *θ* is a scaling factor. When *θ* = 0, this yields the same model estimate of HIV prevalence in pregnant women as the second definition, but as *θ* increases above 0, the effect of the recency adjustment becomes more strongly positive.

### Scenarios and sensitivity analyses

The ‘default’ scenario refers to the set of assumptions used in the calibration of the MicroCOSM model to available HIV prevalence and HIV programme data. It represents the most realistic assessment of what has happened in South Africa up to 2017, taking into account the HIV programmes that have been introduced, the changes in sexual behaviour that have occurred and the changes in contraceptive uptake over time (see Table 1).

To understand the factors that contribute to antenatal bias, we consider a number of counterfactual scenarios:

- ‘No change’ scenario: Assuming no HIV interventions, no change in patterns of contraceptive uptake or breastfeeding, and no changes in antenatal testing algorithm (antenatal testing is assumed to be 100% specific);
- ‘No change in breastfeeding’ scenario: Assuming no difference in breastfeeding durations when comparing HIV-positive mothers and HIV-negative mothers, and no change in average breastfeeding durations over time;
- ‘No change in antenatal testing’ scenario: Assuming no dropping of confirmatory HIV testing from 1997 and subsequent antenatal surveys (i.e. no addition of anticipated false-positive test results when calculating *α*(*x*,*t*));
- ‘No pregnancy effect on transmission’ scenario: Assuming pregnancy has no effect on either the male-to-female or female-to-male transmission probability per sex act;
- ‘Constant condom use’ scenario: Assuming no change in condom use over time;
- ‘No change in hormonal contraceptive use’ scenario: Assuming no change over time in hormonal contraceptive uptake among young women (ages 15–24); and
- ‘Proportional racial representation in public antenatal clinics’ scenario: Assuming all pregnant women are equally likely to use public antenatal facilities, regardless of their race.

No recalibration of the model to the HIV prevalence data is performed in any of the counterfactual scenarios.

### Comparisons based on the Thembisa mode1

To assess the practical significance of the different assumptions about antenatal bias, we compare the results obtained using the Thembisa model. The Thembisa model is a deterministic HIV and demographic model developed for South Africa. The model is fitted to antenatal HIV prevalence data and household survey data, separately for each of South Africa’s nine provinces, using a Bayesian procedure. A full description of the model is provided elsewhere [27, 52], and section 2 of the S1 File describes the calibration procedure under each of three approaches: (a) assuming antenatal bias is constant when using the unadjusted definition, (b) assuming antenatal bias is constant when using the adjusted definition, and (c) assuming antenatal bias is constant when using the recency-adjusted definition. Version 4.2 of the model used the adjusted definition of antenatal bias (although on a logit scale rather than a log scale) when calibrating to the antenatal surveillance data, i.e. assuming that there is a constant difference (on the logit scale) between modelled HIV prevalence in sexually experienced women and HIV prevalence in antenatal surveys, after controlling for the effect of HIV on fertility. We compare the results from version 4.2 with the results obtained if we instead assume constancy of the unadjusted and recency-adjusted measures of antenatal bias, for each of the nine provinces. We also compare these results with the results obtained when calibrating the model only to household survey data (i.e. excluding antenatal HIV data and thus avoiding any assumptions about antenatal bias).

## Results

Fig 1 shows the MicroCOSM model estimates of HIV prevalence trends, in pregnant women and in the population aged 15–49, with comparisons to South African survey data. HIV prevalence in pregnant women increased rapidly during the 1990s, but stabilized after 2004. In contrast, HIV prevalence in the 15–49 population has increased steadily, with no sign of stabilization in the period up to 2017.

### Comparison of unadjusted and adjusted definitions of antenatal bias

**Fig 2:**
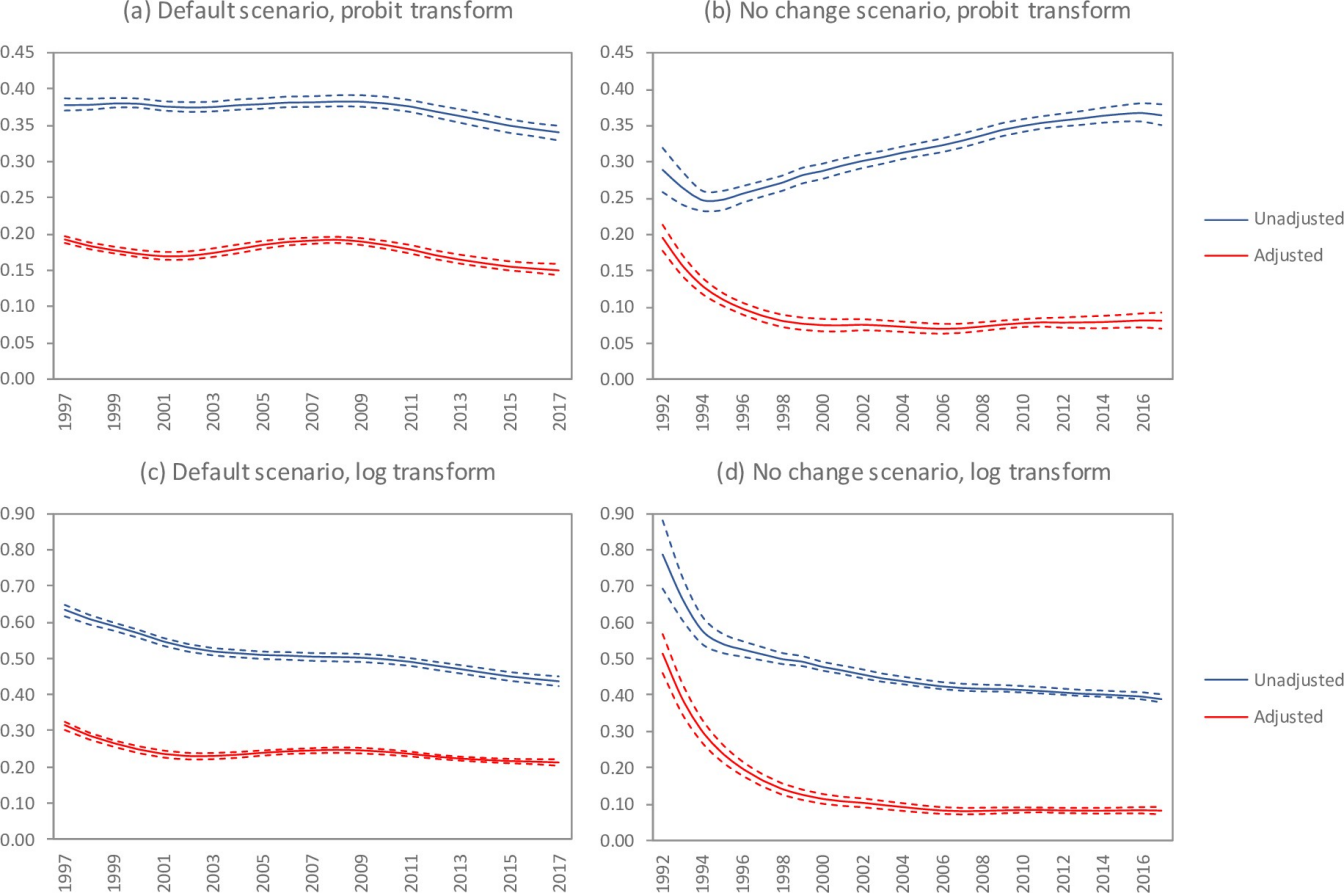
Comparison of different measures of antenatal bias. Solid lines represent means and dashed lines represent 95% confidence intervals. Results for the default scenario are not shown for the pre-1997 period because changes in assumed test specificity imply a discontinuity in antenatal bias, and because these data are not used in model calibration. However, earlier results are shown in the ‘no change’ scenario as this scenario assumes no change in antenatal test specificity (100%).

In the default scenario, antenatal bias appears to have changed slightly over time on the probit scale (Fig 2A). Using the unadjusted measure of antenatal bias, antenatal bias appears to have been roughly stable up to 2010, then gradually declined. Using the adjusted measure of antenatal bias, antenatal bias declined slightly over the 1997–2002 period, then increased slightly up to 2008 before declining again. On the log scale, however, the antenatal bias appears to have followed a more steady decline over time (Fig 2C). This decline in antenatal bias over time is also apparent when considering the counterfactual scenario in which there is assumed to have been no changes in sexual behaviour or contraception over time and no HIV interventions (Fig 2B and 2D). However, on the probit scale the unadjusted measure of antenatal bias increased steadily after 1995 (Fig 2B), as the probit transformation tends to inflate differences in prevalence as HIV prevalence increases (S16a Fig in S1 File). The antenatal bias in the ‘no change’ counterfactual scenario is substantially lower than that in the default scenario, although in the case of the unadjusted definition there has been a degree of convergence in antenatal bias in recent years when comparing the default and ‘no change’ scenarios.

### Factors accounting for antenatal bias and change in antenatal bias over time

**Fig 3:**
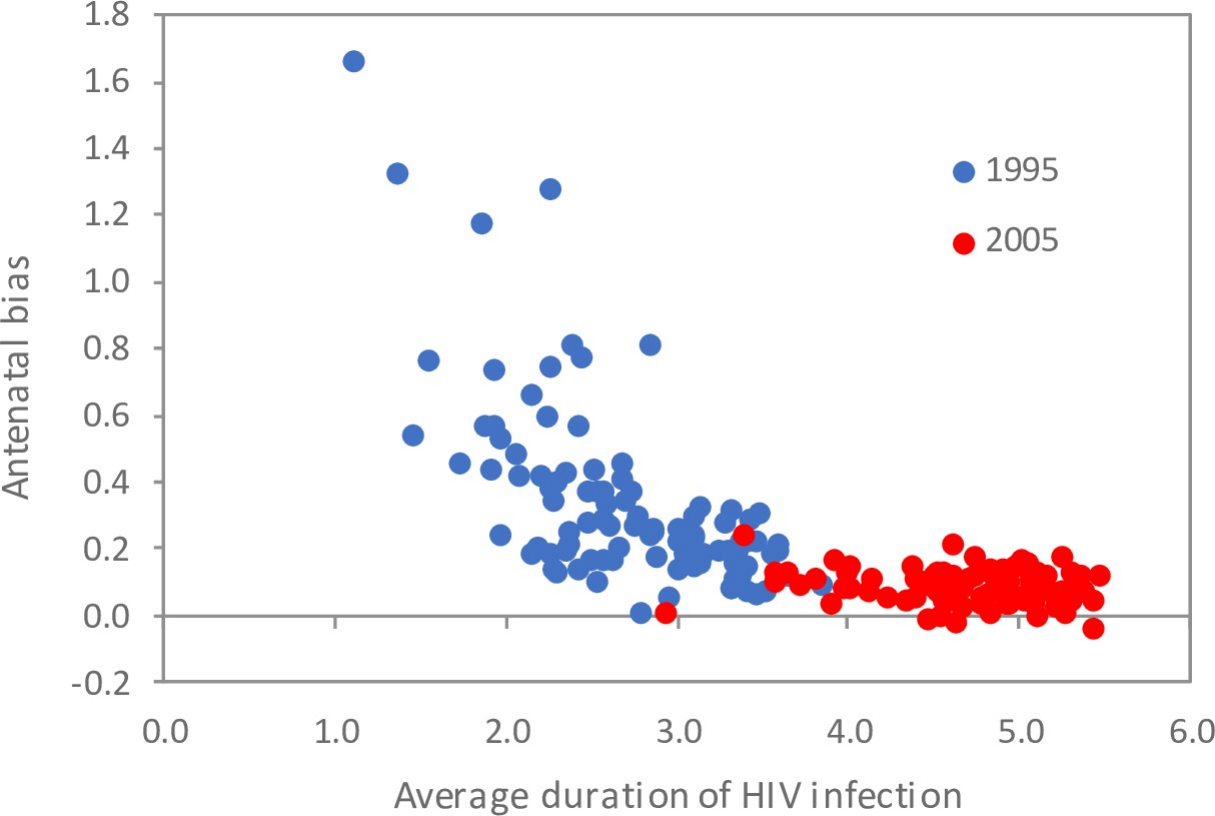
Correlation between average duration of HIV infection in pregnant HIV-positive women and antenatal bias. Antenatal bias is calculated using the adjusted definition (on a log scale) in the ‘no change’ scenario. Each dot represents the results from a model simulation with a different input parameter combination. Different parameter combinations were sampled from distributions that were considered plausible, and the 100 parameter combinations that yielded the best fit to the HIV prevalence data were used to generate these 100 model simulations. The average duration of HIV in pregnant women was calculated 5 years after the antenatal bias to provide a more sensitive metric of the epidemic growth rate than would be obtained if average duration was calculated in the same year as the antenatal bias.

To gain a better understanding of the declining trend in antenatal bias in the ‘no change’ scenario (Fig 2D), we assessed the correlates of antenatal bias (adjusted definition, log scale) in 1995 (early epidemic) and 2005 (mature epidemic). The level of antenatal bias in 1995 was strongly negatively correlated with the average duration of HIV in pregnant HIV-positive women in 2000, a measure of the rate of epidemic expansion in the early epidemic (r = -0.65, Fig 3). This means that when the epidemic was expanding rapidly (short average duration of infection), antenatal bias was substantial, probably because the HIV-positive women who were recently infected were more likely to be represented in the pregnant population (by virtue of having had recent unprotected sex). The relationship between antenatal bias and the average duration of infection appears to be non-linear, suggesting that antenatal bias was driven mainly by infections of short duration.

**Fig 4:**
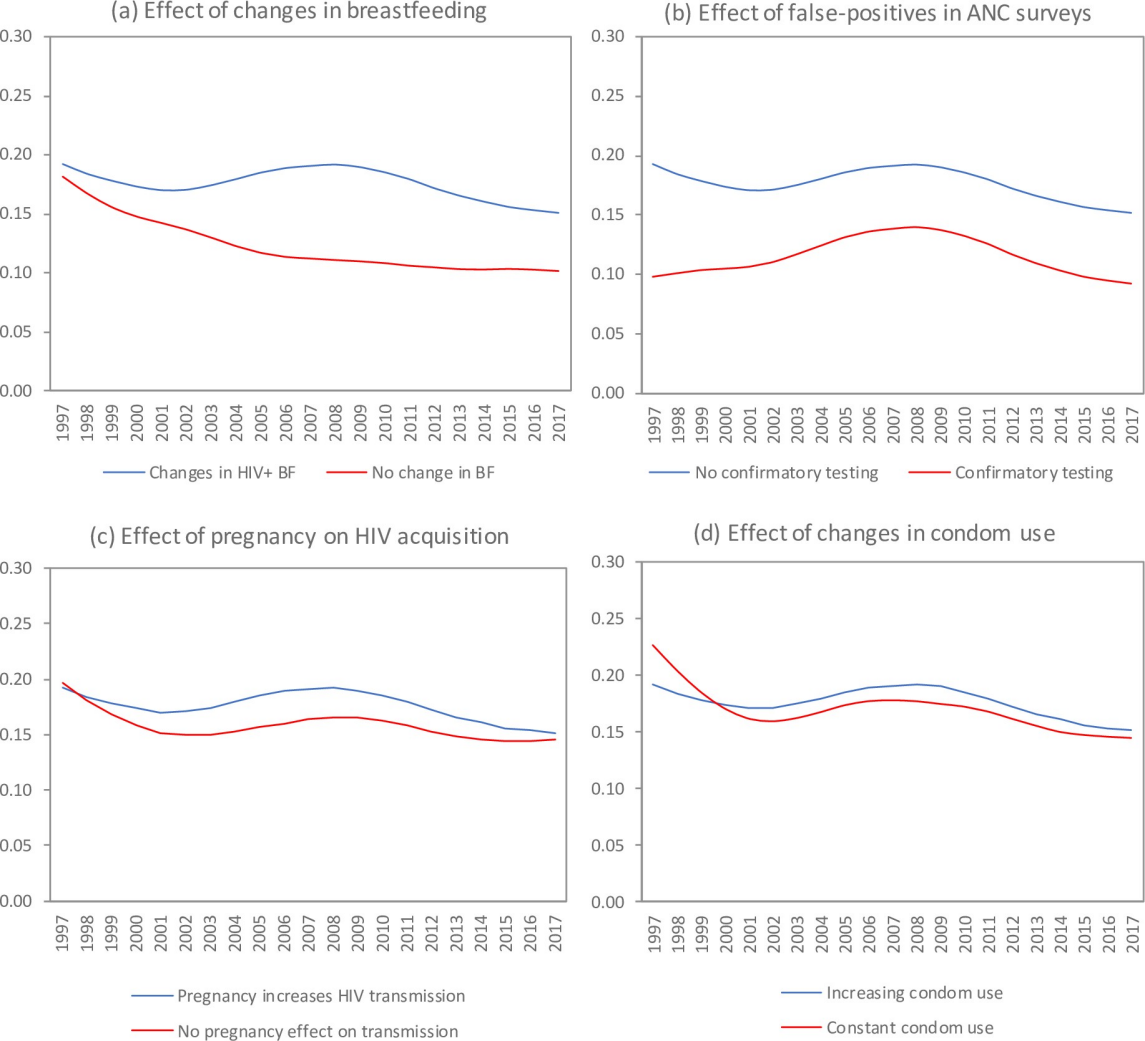
Factors accounting for antenatal bias and change in antenatal bias over time. Antenatal bias is calculated using the adjusted definition (on a probit scale). In all panels, the blue line corresponds to the default scenario. Solid lines represent means from 100 simulations. ANC = antenatal clinic, BF = breastfeeding.

To better understand the higher antenatal bias in the default scenario (relative to the ‘no change’ scenario) and the change in antenatal bias after the early epidemic growth phase, we considered a number of counterfactual scenarios (Fig 4). Antenatal bias would have been significantly lower if HIV-positive women had breastfed for the same duration as HIV-negative women (Fig 4A), which would have led to fewer births in HIV-positive women due to longer lactational amenorrhoea. The difference in bias (relative to the default scenario) was small in 1997 when relatively few HIV-positive women knew they were HIV-positive, then increased as HIV testing and PMTCT programmes were rolled out, and finally declined around the time of the withdrawal of free formula milk from PMTCT programmes in 2011. Antenatal bias would also have been significantly lower if there had been no false positive results in the antenatal surveys (Fig 4B); the difference in bias (relative to the default scenario) was particularly substantial in 1997, when HIV prevalence was relatively low. A less important determinant of the antenatal bias was the effect of pregnancy on HIV acquisition (Fig 4C); if the rate of HIV acquisition in pregnant women was the same as in non-pregnant women, the correlation between pregnancy and HIV would have been weaker and the antenatal bias would have been smaller. The effect of the change in hormonal contraceptive use in young women on antenatal bias was negligible, although the antenatal bias was slightly higher among 15–24 year olds in the counterfactual scenario in which hormonal contraceptive uptake was assumed to remain constant over time (S18 Fig in S1 File).

The effect of changes in condom use on antenatal bias appears modest (Fig 4D). In the absence of any change in condom use, the antenatal bias would have been higher than that in the default scenario up to 2000, but lower thereafter. The association between increased general condom use and increased antenatal bias in the post-2000 period is to be expected, because as more women used condoms consistently, the antenatal surveys became less representative of the general female population and the consistent condom users who were missed by the antenatal survey were at lower risk of HIV infection, which implies that the antenatal data were more likely to overstate HIV prevalence in the female population. The association between increased condom use and reduced antenatal bias in the pre-2000 period is probably due to an ‘early adopter’ effect in the early stages of condom promotion programme: the model assumes that higher risk women (sex workers and women in short-term relationships) were most likely to start using condoms, and thus in the early stages of the condom promotion programme there appeared to be a positive relationship between condom use and HIV prevalence. Only in the longer term, as the cumulative benefits of consistent condom use became more substantial, did the relationship between condom use and HIV prevalence switch to being negative.

### Age differences in antenatal bias, and effect of recency adjustment

**Fig 5:**
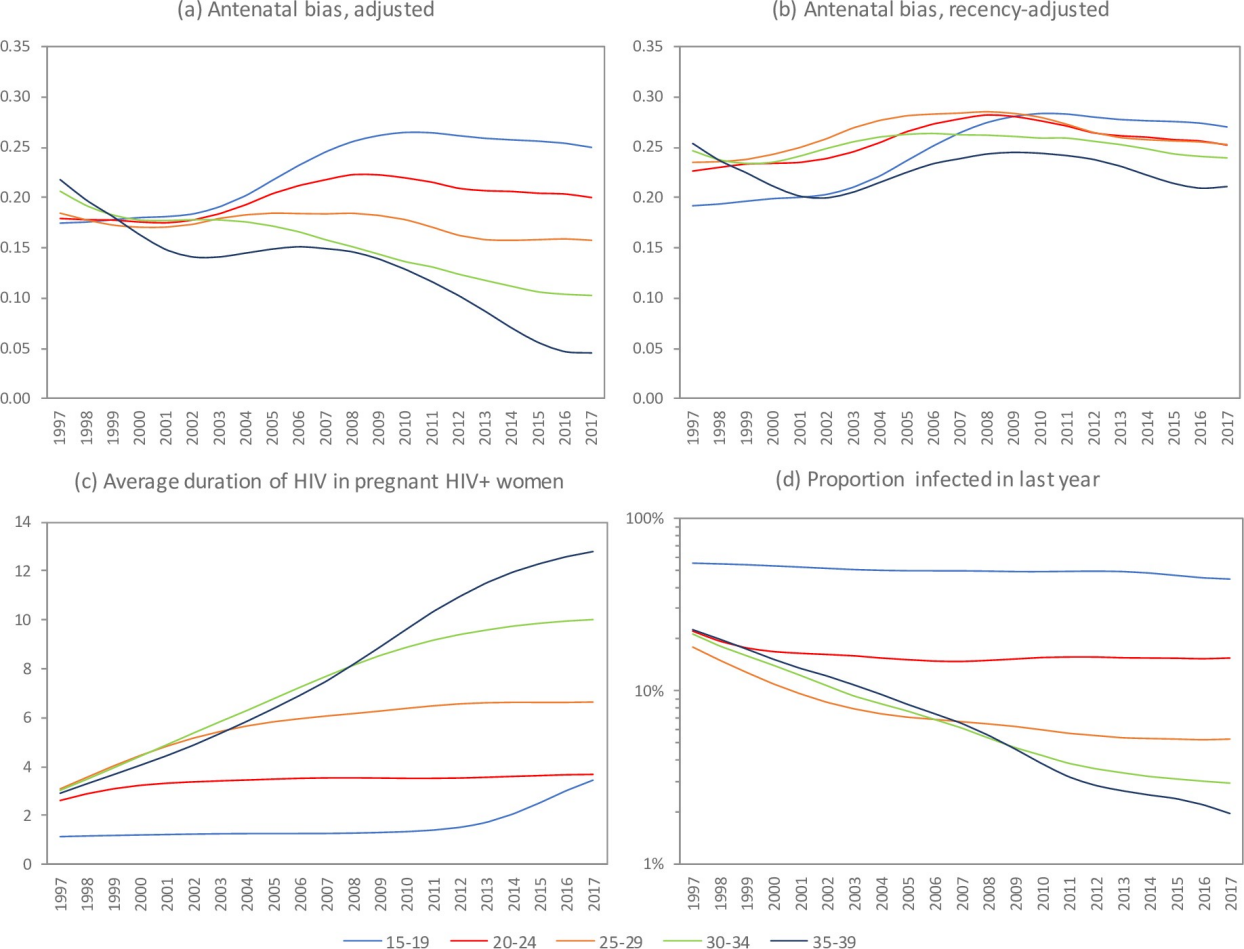
Age differences in antenatal bias and average duration (in years) of HIV infection. Antenatal bias is calculated using the adjusted definition (panel a) and the recency-adjusted definition (panel b), both on a probit scale. Average duration of HIV in pregnant HIV-positive women increases after 2012 due to the survival of vertically-infected girls into adolescence as the epidemic matures (panel c).

When considering the adjusted definition of antenatal bias, there were substantial differences in antenatal bias by age, and trends in antenatal bias over time also differed substantially by age (Fig 5A). Around 2000, levels of antenatal bias were similar across the five age groups considered, but in subsequent years, there was a trend toward increasing antenatal bias in the 15–24 age group and a trend towards steeply declining antenatal bias in the 30–39 age group. The antenatal bias appeared to be inversely associated with the average duration of HIV infection in pregnant HIV-positive women, with the average duration of HIV infection in pregnant women increasing over time in older women but remaining relatively stable in younger women (Fig 5C). This led us to hypothesize that the recency-adjusted measure of antenatal bias would yield more stable estimates of antenatal bias that differ less dramatically between age groups. Estimates of the fraction of HIV infections in pregnant women that were recently acquired (the *λ*(*x*,*t*) term in the recency adjustment formula) are shown in Fig 5D: the fraction is estimated to have remained stable, at around 50% and 20% in the 15–19 and 20–24 age groups respectively, but in women aged 25 and older the fraction is estimated to have declined from around 20% in 1997 to 5% or less in recent years. Setting the *θ* scaling factor to 0.04 minimized the standard deviation of the recency-adjusted bias estimates over the 1997–2017 period (0.024 compared to 0.046 with the adjusted definition), and yielded greater similarity in antenatal bias estimates across age groups (Fig 5B).

### Geographical differences in antenatal bias

**Fig 6:**
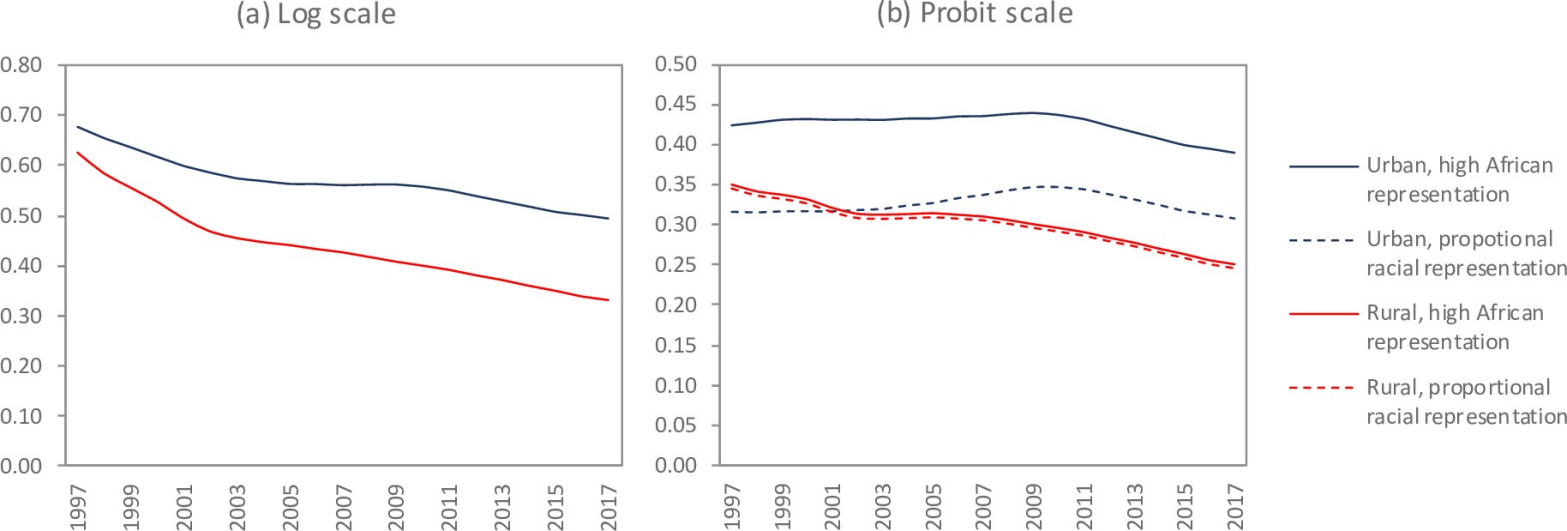
Urban-rural differences in antenatal bias (unadjusted definition). In the default scenario (solid lines), pregnant women who are African are more likely to use public antenatal facilities than those who are not African. In the counterfactual scenario (dashed lines), all pregnant women are assumed to have the same probability of public antenatal clinic use, regardless of their race.

Antenatal bias was significantly greater in urban areas than in rural areas (Fig 6A). We hypothesized that these urban-rural differences were largely due to differences in the racial composition of urban and rural populations, combined with racial differences in use of public antenatal services. To test this hypothesis, we considered a counterfactual scenario in which all pregnant women were assumed to be equally likely to use public antenatal services, regardless of their race (Fig 6B). In this scenario, antenatal bias in urban areas was substantially lower than in the default scenario, reflecting the lower HIV prevalence in racial minorities (white, Asian and mixed-race women), who are less likely to use public antenatal clinics. However, antenatal bias in rural areas was almost identical in the counterfactual and default scenarios, as racial minorities comprise a relatively small fraction of the rural population.

### Testing the effect of different antenatal bias assumptions

**Fig 7:**
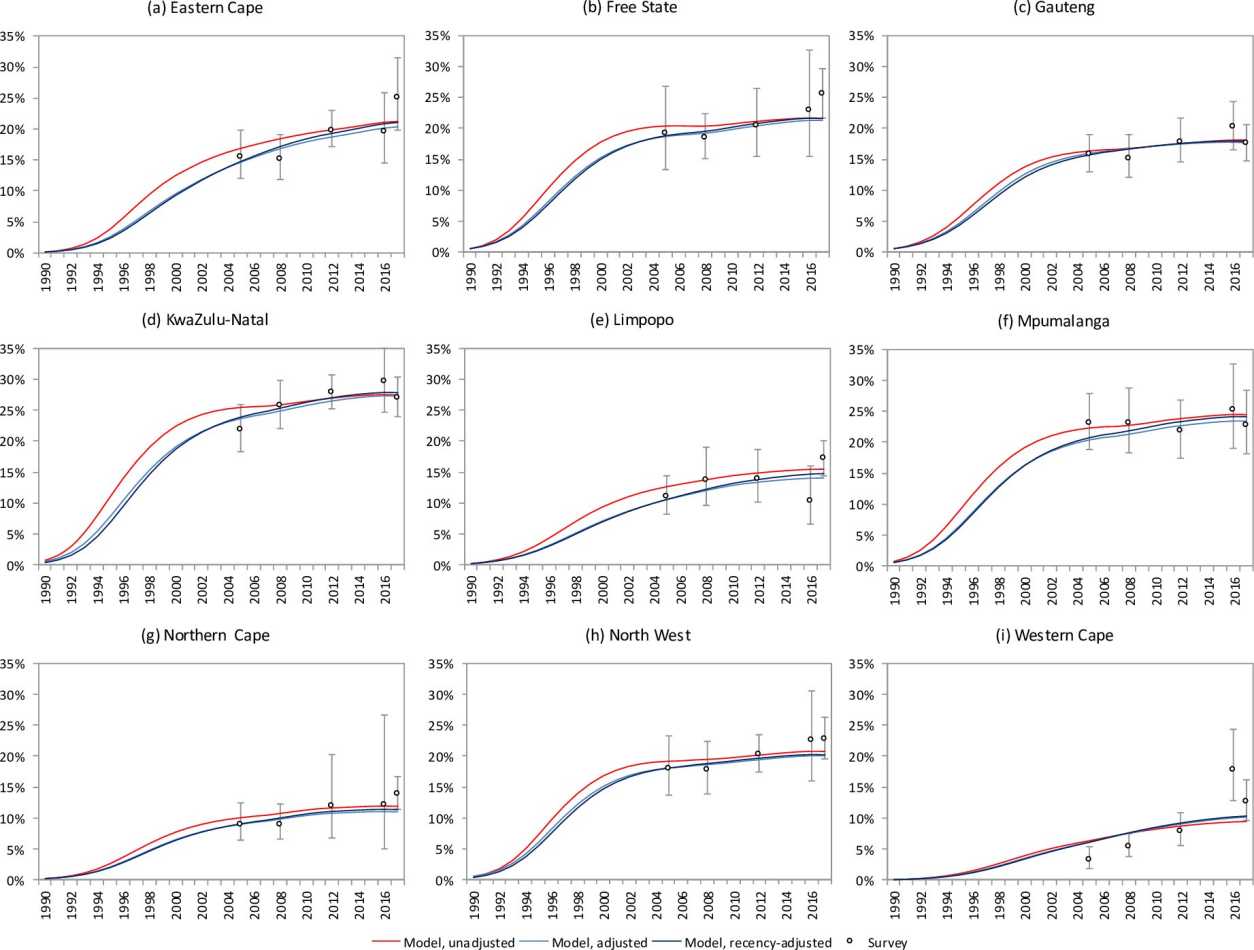
HIV prevalence in population aged 15–49, as estimated by the Thembisa model under different assumptions about constant antenatal bias.

**Fig 8:**
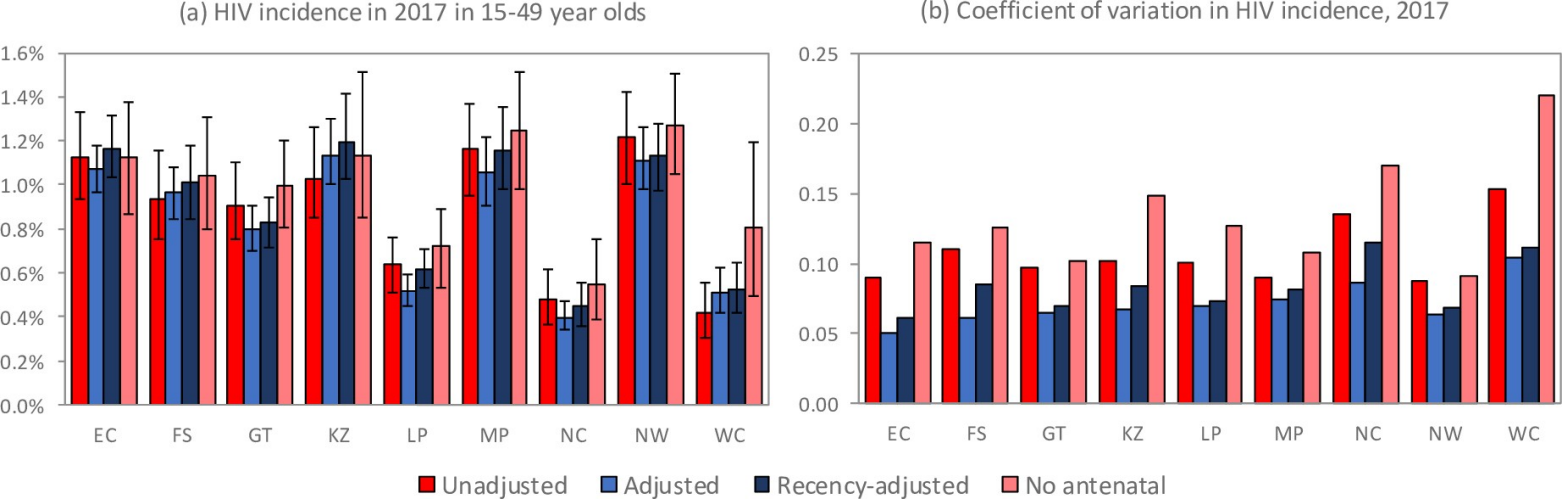
HIV incidence in 2017, as estimated by the Thembisa model under different assumptions about constant antenatal bias. The coefficient of variation (panel b) is defined as the standard deviation of the model incidence estimates divided by the mean model estimate. EC = Eastern Cape, FS = Free State, GT = Gauteng, KZ = KwaZulu-Natal, LP = Limpopo, MP = Mpumalanga, NC = Northern Cape, NW = North West, WC = Western Cape.

When the Thembisa model was calibrated to provincial HIV prevalence data using the three different definitions of antenatal bias, results were roughly similar for adjusted and recency-adjusted definitions of antenatal bias, but substantially different when using the unadjusted definition. Using the unadjusted definition of antenatal bias, Thembisa consistently estimated higher levels of HIV prevalence in the earlier stages of the HIV epidemic, in all provinces (Fig 7), and 95% confidence intervals around HIV incidence estimates were consistently wider than those obtained using the adjusted definition of antenatal bias (Fig 8 and S4 Table in S1 File). Although the use of the recency-adjusted definition of antenatal bias in the model calibration had only a modest impact on the results of the Thembisa model, when compared to the results obtained using the adjusted definition of antenatal bias, the use of the recency adjustment led to slightly lower estimates of HIV prevalence in the early HIV epidemic but slightly higher estimates of HIV prevalence in more recent years (Fig 7 and S13 Fig in S1 File). The recency-adjusted estimates of HIV incidence in 2017 were on average 8% higher (range 2–19%) than those obtained using the adjusted definition of antenatal bias (Fig 8A and S13d Fig in S1 File), and recency-adjusted incidence estimates had slightly greater coefficients of variation (Fig 8B). The difference between recency-adjusted and adjusted incidence estimates was much more marked in the 25–49 age group than in the 15–24 age group (S13g and S13h Fig in S1 File), meaning that youth account for a lower proportion of new infections in the 15–49 age group, when using the recency adjustment (S13i Fig in S1 File). Thembisa estimates of HIV prevalence and incidence were not sensitive to the choice of transformation in the antenatal bias definition: logit, log and probit transformations yielded similar estimates (S17 Fig in S1 File).

When the Thembisa model was calibrated only to the household survey data (not including antenatal data), estimates of HIV incidence in 2017 were mostly similar to those obtained when including the antenatal data, except in the Western Cape (Fig 8A). However, coefficients of variation around the HIV incidence estimates were consistently higher than those obtained when including the antenatal survey data in the calibration (Fig 8B). Further comparisons of HIV prevalence and incidence estimates in earlier years are shown in S14 and S15 Figs in S1 File.

## Discussion

Even in countries such as South Africa, where HIV prevalence has been measured in several national household surveys, the inclusion of antenatal HIV prevalence data remains important in improving the precision and accuracy of recent HIV estimates. However, biases in South African antenatal HIV prevalence data are complex and arise for a number of reasons. In using antenatal HIV prevalence data to assess trends in HIV prevalence and incidence, it is particularly important to consider the ways in which this bias may change over time. Previous modelling studies have demonstrated how bias may change over time due to differences in the age distribution of pregnant women and women in the general population [16], the effect of HIV on fertility [53], and changes in age at sexual debut [17]. In this modelling study we consider a number of further factors that may contribute to changes in antenatal bias over time, such as changes in recency of HIV infection in pregnant women, changes in condom use and hormonal contraception, and changes in breastfeeding by HIV-positive mothers.

The recency of HIV infection in pregnant women is an important source of bias that has not previously been assessed. When the average duration of infection is short, HIV-positive women are relatively more likely to be represented in samples of pregnant women, as recent pregnancy and recent HIV acquisition are both strongly dependent on recent unprotected sex. When the average duration of HIV infection is long, the recently-infected women make up a relatively small proportion of total HIV-positive women, and hence the correlation between HIV infection and pregnancy is not as strong. This dynamic explains why adjusted antenatal bias tends to decline over the course of an HIV epidemic, as HIV prevalence increases and as average durations of infection increase. It also explains why in more mature epidemics there are substantial age differences in antenatal bias, as younger HIV-positive women are much more likely to have been recently infected than older HIV-positive women. The same age differences are not apparent early in the epidemic because in the early stages of the epidemic, most HIV-positive women are likely to have been recently infected, regardless of their age. Most published data on differences in fertility between HIV-positive women and HIV-negative women are from mature HIV epidemics [54, 55], and our results suggest that relationships between HIV and fertility are likely to be different in early-stage epidemics, when HIV incidence rates are high. Recent moves to introduce rapid testing for recent infection [56] could be important in assessing antenatal bias, as such tests, when conducted in pregnant women, could be used to determine the proportion of infections that are recently acquired.

We have proposed an alternative measure of antenatal bias, which adjusts for the recency of HIV infection, and have shown that this roughly halves the standard deviation of the antenatal bias terms (by age and by year) relative to that calculated when adjusting only for age, sexual experience and HIV stage. Although this adjustment does not completely stabilize the bias (due to factors discussed below), it does imply that there would be less error in assuming that duration-adjusted antenatal bias terms are constant over time and by age. Using this recency-adjusted measure of antenatal bias should ensure that models produce better estimates of HIV prevalence trends and HIV prevalence patterns by age.

Changes in breastfeeding in HIV-positive women are another important explanation for changes over time in antenatal bias, since lactational amenorrhoea is a major determinant of fertility [18]. Prior to the introduction of PMTCT programmes, few HIV-positive mothers knew their HIV status, and most would therefore have breastfed for durations similar to HIV-negative mothers, implying minimal bias. However, WHO guidelines on breastfeeding in the early 2000s promoted replacement feeding as the preferred feeding method for HIV-positive mothers, and it was only in 2006 that WHO guidelines started to promote exclusive breastfeeding and longer durations of breastfeeding as the ‘default’ recommendation for HIV-positive mothers [57]. Most African countries have broadly followed these guidelines, implying an increase in antenatal bias as early PMTCT programmes were introduced, followed by a reduction in antenatal bias as countries switched to promoting exclusive breastfeeding by HIV-positive mothers. However, countries have differed in the timing of these changes: in South Africa the withdrawal of free formula milk occurred only in 2011 [58], and Botswana has only recently switched away from promoting replacement feeding.

Although changes in condom use have probably caused some change in antenatal bias in the South African setting, our simulations suggest that the effect is likely to be modest. In the early stages of the behaviour change, the ‘early adopters’ of condoms tended to be higher-risk women, and their lower likelihood of falling pregnant implied a reduction in antenatal bias. However, in the longer term, the cumulative benefits of consistent condom use became more substantial, and women who used condoms consistently had a lower risk of HIV as well as a lower chance of falling pregnant, hence an increase in antenatal bias. The effect of changes in condom use on antenatal bias is likely to have been minimal in most other sub-Saharan African settings, where the proportions of women who report using condoms for contraceptive purposes tend to be less than 5% [59], in contrast to the level of 15% in South Africa in 2016 [36].

Our simulations suggest that changes in levels of hormonal contraceptive use also have almost no effect on the level of antenatal bias. To the extent that increases in hormonal contraceptive use among young women are associated with a modest increase in HIV risk, a small reduction in antenatal bias might be expected to follow an increase in hormonal contraceptive use, because this would increase HIV prevalence in women who are not at risk of falling pregnant. However, this finding is dependent on the assumption that injectable contraceptive use increases women’s susceptibility to HIV. Although this assumption is supported by observational evidence [43], only one randomized clinical trial has been conducted to assess this question; the trial found that HIV incidence rates were higher in women using injectable contraception than in women using other contraceptive methods, but the difference was not significant [60]. The trend toward declining hormonal contraceptive use among young South African women is unusual; in most other sub-Saharan African countries, there have been substantial increases in hormonal contraceptive use over the last two decades [59].

There are several other factors that are important in explaining antenatal bias, and although these factors do not necessarily account for changes in antenatal bias over time, they may nevertheless be important in understanding inter-regional differences in antenatal bias. The substantial urban-rural differences in antenatal bias in South Africa are explained to a large extent by racial differences in use of public antenatal facilities and urban-rural differences in the racial distribution of South Africa’s population, a legacy of apartheid policies that restricted settlement patterns. The results of this study are consistent with a previous modelling study that found antenatal bias in South Africa to be substantially higher in the highly urbanized provinces (Gauteng and Western Cape) than in the rest of the country [27]. Although this dynamic is probably unique to South Africa, it is possible that there may be socio-economic differences in use of antenatal care in other settings that similarly affect antenatal bias. In many countries a substantial proportion of pregnant women do not access antenatal care, and these women are typically poorer and less likely to be married than those who seek antenatal care [61]. To the extent that utilization of antenatal care differs substantially across regions, and to the extent that HIV risk differs between attenders and non-attenders, inter-regional variation in antenatal bias may be expected.

Differences in HIV testing algorithms between antenatal surveys and household surveys are another potential source of bias. In South Africa, the antenatal surveys that have been conducted annually since 1997 have relied on a single ELISA test per subject, and in the absence of confirmatory testing, some exaggeration might be expected due to false positive reactions [62]. However, the extent of this exaggeration is highly uncertain; in an analysis of HIV testing data from 20 different Demographic and Health Surveys, Fishel and Garrett [62] found that the fraction of initially reactive specimens that were confirmed positive on a second ELISA varied between 31% and 97%. Our assumption of 97.7% specificity (equivalent to a positive predictive value of about 95% when prevalence is 30%) might therefore appear optimistic, although in a recent analysis of 2017 South African antenatal survey data, test specificity of close to 100% was estimated (Adrian Puren, personal communication). False-positive reactions are likely to cause a relatively large antenatal bias when HIV prevalence is low (i.e. in early epidemics and in the youngest age groups). In recent years, many African countries have discontinued antenatal sentinel surveillance and are increasingly relying on routine programmatic data on HIV testing at antenatal clinics for PMTCT purposes [5, 9], which potentially introduces a different kind of bias. Most PMTCT programmes require two or more positive rapid tests before an HIV diagnosis is made, and concern has been raised over the poor quality of testing in some settings, which may result in HIV being under-diagnosed (i.e. the problem is mainly one of false negative reactions) [63]. A more serious problem is that women who disclose that they have already been diagnosed might not be included in the PMTCT prevalence calculation (because they are not tested), or HIV-negative women may be double-counted (because of guidelines to repeat HIV testing in pregnant women who were HIV-negative at their first antenatal visit) [28]. Such errors may render routine HIV prevalence data from PMTCT programmes meaningless.

Our comparison of the effect of different antenatal bias assumptions in the Thembisa calibration process suggests that model results are sensitive to assumptions about antenatal bias. The unadjusted approach to defining antenatal bias is particularly problematic, yielding HIV incidence and prevalence estimates that are too high in the early stages of the HIV epidemic. The unadjusted approach, because it does not require the use of age-specific antenatal prevalence data, also produces less precise HIV incidence estimates, with confidence interval widths being up to twice as wide as those obtained using the adjusted approach (Fig 8B). The improvement in precision and accuracy, under the adjusted approach, is important for the purpose of assessing whether incidence reduction targets have been achieved. Previously published HIV epidemic estimates based on the EPP model [64] and Global Burden of Disease Study [65] overstated the early growth and peak of the epidemic in South Africa, and consequently also overstated the numbers of AIDS deaths in South Africa [66]. These estimates used the unadjusted approach for estimating the course of the South African HIV epidemic from antenatal surveillance data, which may explain the overestimation of historical epidemic growth and AIDS deaths.

Although the recency adjustment to the antenatal bias did not substantially change the Thembisa model estimates, it did lead to a slightly later epidemic peak, with HIV prevalence and incidence being estimated to be slightly higher in recent years than estimated using the adjusted definition of antenatal bias. HIV incidence rates in 2017 were between 2% and 19% higher when using the recency-adjusted approach, which is potentially important when evaluating whether targets for epidemic control have been achieved [2]. Most significantly, the recency adjustment leads to a shift in the age distribution of HIV incidence (relatively more incident HIV in older adults). However, incidence estimates are slightly less precise under the recency-adjusted approach than under the adjusted approach (Fig 8B), as the bias under the recency-adjusted approach is itself a function of the incidence rate.

A limitation of this analysis is that MicroCOSM, the source of our simulated data, does not perfectly match observed age patterns of HIV prevalence in pregnant women and women in the general population, nor does it perfectly match observed trends in HIV prevalence over time (S1 and S2 Figs in S1 File). We cannot claim to have characterized antenatal bias or changes in antenatal bias in South Africa with perfect accuracy; our objective is only to gain qualitative insights into the factors that might plausibly account for antenatal bias in South Africa. Parameters such as the specificity of the ELISA used in the antenatal surveys and the effect of injectable contraception on women’s susceptibility to HIV are difficult to estimate from available data sources, and estimates of antenatal bias may be sensitive to changes in these assumptions. Due to the complexity of the MicroCOSM calibration process, it was not feasible to consider the uncertainty around these parameters in the model calibration. There is also uncertainty regarding the effect of HIV on fertility and–more significantly–the effect of ART on fertility [67]. We have used the same assumptions about HIV and ART effects on fertility in the adjusted definitions as are assumed in the MicroCOSM model, to ensure internal consistency within the simulated population. However, to the extent that the adjusted definitions are applied to ‘real’ data, from populations in which the true effects of HIV and ART on fertility are unknown, this uncertainty remains a concern.

Another limitation is that–although we have modelled the effect of other STIs on HIV transmission–we have not modelled STI effects on infertility. An early modelling study suggested that infertility caused by STIs could lead to a change in antenatal bias over time, with the highest-risk women who suffer the most from infertility acquiring HIV early in the epidemic [53]. However, most STI-associated infertility occurs in older women [68], and this might therefore not be a major source of bias.

These results suggest that in the South African setting, antenatal bias has not remained stable over time. This finding holds across a number of alternative definitions of antenatal bias. Importantly, the finding holds even in a ‘no change’ scenario, which suggests findings may be relevant to other African settings, where HIV interventions and changes in contraceptive patterns may have been different from those in South Africa. These results have important implications for efforts to estimate HIV incidence trends from antenatal HIV prevalence data, which are critical in light of global targets for HIV epidemic control.

## Supporting information

S1 Dataset HIV prevalence data used in model calibration. (XLSX)

S1 File (DOCX)
