# Supplementary material for: Challenges in estimating HIV prevalence trends and geographical variation in HIV prevalence using antenatal data: Insights from mathematical modelling
Source: PLoS One. 2020 Nov 20;15(11):e0242595. doi: 10.1371/journal.pone.0242595 (PMC7679018; doi:10.1371/journal.pone.0242595)
Supplement: S1 File — (DOCX) [file pone.0242595.s002.docx]

Challenges in estimating HIV prevalence trends and

geographical variation in HIV prevalence using antenatal data:

insights from mathematical modelling

Table of contents

[1. Calibration of the MicroCOSM model 2](#_Toc54125044)

[2. Calibration of the Thembisa model 4](#_Toc54125045)

[2.1 Method 1: Unadjusted definition of antenatal bias 5](#_Toc54125046)

[2.2 Method 2: Adjusted definition of antenatal bias 7](#_Toc54125047)

[2.3 Method 3: Recency-adjusted definition of antenatal bias 9](#_Toc54125048)

[2.4 Results of calibration 10](#_Toc54125049)

[2.5 Sensitivity analysis: Calibration without antenatal HIV prevalence data 22](#_Toc54125050)

[2.6 Sensitivity analysis: Alternative transformations 24](#_Toc54125051)

[3. Additional MicroCOSM outputs 26](#_Toc54125052)

[References 28](#_Toc54125053)

# **1. Calibration of the MicroCOSM model**

Figure S1 shows the MicroCOSM calibration to antenatal survey HIV prevalence data. Overall, the model estimates of HIV prevalence in pregnant women using public antenatal clinics are reasonably consistent with the antenatal survey data (after allowing for some false-positive reactions in the survey data). However, there are some inconsistencies when examining individual age groups: the model slightly under-estimates HIV prevalence in pregnant women in the 35-39 age group, and in the period after 2005, the model slightly over-estimates HIV prevalence in pregnant women aged 25-29. The model is also less consistent with the antenatal surveys conducted prior to 1997 (which have not been included in the calibration because the early surveys did not follow a standard sampling protocol). Although the model fits to the antenatal survey data are not ideal, it should be noted that exact consistency is very difficult to achieve when the antenatal bias processes are being modelled mechanistically, rather than through an arbitrary bias parameter (as in the Thembisa model, described in the following section).

Figure S2 shows the MicroCOSM calibration to the HIV prevalence data collected by the Human Sciences Research Council (HSRC) in national household surveys. Model results are generally quite consistent with the age- and sex-specific HIV prevalence data in each survey year, although the age pattern of HIV prevalence in men is irregular in some of the HSRC surveys.

Figure S1: HIV prevalence in pregnant women attending public antenatal clinics

Model results have been adjusted to reflect the antenatal biases described previously (note that the antenatal bias changes in 1997 due to changes in antenatal testing protocols; surveys in 1997 and subsequent years did not include confirmatory testing and some exaggeration due to false positive test results is therefore expected). Model results are the average results generated using the 100 best-fitting parameter combinations.

Figure S2: HIV prevalence in the general population

Model results are the average results generated using the 100 best-fitting parameter combinations.

# **2. Calibration of the Thembisa model**

The Thembisa model is calibrated to a number of data sources. For the purpose of this comparison of the results obtained when applying the model to each of the nine provinces of South Africa, we consider only two data sources: the HIV prevalence data in household surveys and the HIV prevalence in antenatal clinic surveys. The calibration procedure is described more fully in section 8 of the Thembisa provincial modelling report [1]. This section provides a brief overview of the calibration procedure, with more detail on the approach to defining the likelihood in respect of the antenatal survey data.

Briefly, Thembisa is calibrated using a Bayesian approach. In the first step, we specify prior distributions around 8 of the model parameters, each prior distribution representing the uncertainty around the relevant parameter, based on prior knowledge regarding HIV epidemiology in South Africa. In the second step, we specify a likelihood function to represent how well the Thembisa model fits the observed levels of HIV prevalence in the relevant province, for a given set of input parameters. In the third and final step, we numerically simulate the posterior distribution (which is proportional to the product of the prior distribution and likelihood function) using Incremental Mixture Importance Sampling (IMIS) [2]. The posterior distribution represents the set of input parameters that best fit the available data, subject to the constraints imposed by our prior beliefs.

The prior distributions are all – with one exception – the same as described previously in Table 8.2 of the Thembisa provincial modelling report [1]. The prior distributions on the antenatal bias parameter differ according to the method used to model antenatal bias (as described in the next section).

The likelihood function is defined as the product of two likelihood components: a likelihood for the household survey data, and a likelihood function for the antenatal clinic survey data. The likelihood function for the household survey data is based on the data from five household surveys: the 2005 Human Sciences Research Council (HSRC) survey [3], the 2008 HSRC survey [4], the 2012 HSRC survey [5], the 2016 Demographic and Health Survey (DHS) [6] and the 2017 HSRC survey [7]. The likelihood represents the extent of the consistency with observed HIV prevalence in the 15-24 and 25+ age groups, and the household survey component of the likelihood is defined in the same way regardless of the approach to modelling antenatal bias. The antenatal survey component of the likelihood is based on data from nationally representative surveys conducted every year from 1994 to 2015 and 2017 [8]. The method for calculating this likelihood differs depending on the method for defining antenatal bias, as described in the sections that follow.

## **2.1 Method 1: Unadjusted definition of antenatal bias**

The antenatal likelihood is calculated by comparing model estimates of HIV prevalence in pregnant women and corresponding survey estimates. Suppose that *H_i_*,*_t_*(**φ**) is the model estimate of HIV prevalence adults aged 15-49 in province *i*, in year *t*, where the vector $\mathbf{\varphi}$ represents the values of the model input parameters. The corresponding prevalence of HIV actually measured in the antenatal survey is represented by *y_i_*,*_t_*. It is assumed that if $\mathbf{\varphi}$ is the true set of parameter values, then the difference between the logit-transformed model estimate and the logit-transformed observed prevalence is normally distributed. The mean of this normal distribution represents the extent of antenatal bias, which arises due to a number of factors, such as the exclusion of men and women who aren’t sexually active from the antenatal survey population. The variance of the distribution is assumed to be composed of a ‘survey error’ term, representing the uncertainty around the survey estimate due to binomial variation and cluster variation in the survey, and a ‘model error’ term. More formally, it is assumed that

$\log\left(\frac{y_{i,t}}{1-y_{i,t}}\right)=\log\left(\frac{H_{i,t}\left(\mathbf{\varphi}\right)}{1-H_{i,t}\left(\mathbf{\varphi}\right)}\right)+b_{i}+m_{i,t}+\varepsilon_{i,t}$,

where $b_{i}$ is the antenatal bias parameter in province *i*, $m_{i,t}\sim N\left(0,\sigma_{i}^{2}\right)$ and $\varepsilon_{i,t}\sim N\left(0,\sigma_{i,t}^{2}\right)$. The latter two terms represent the model error and the survey error respectively. The logit transformations ensure that the error terms are closer to normality and that the model error terms are roughly independent of the level of HIV prevalence.

Gamma prior distributions are specified to represent the uncertainty around the antenatal bias parameter, $b_{i}$, separately for each province. The mean of the prior distribution has been chosen based on the average differences (on the logit scale) between the levels of HIV prevalence measured in 15-49 year olds in the 2005, 2008, 2012 and 2017 surveys, and the antenatal survey estimates of HIV prevalence in pregnant women in the corresponding years (Table S1). The standard deviation of the prior distribution is calculated as 15% of the mean, to be consistent with the prior specifications for the other methods for modelling antenatal bias (described below). It is worth noting the logit differences tend to be lower in the more recent survey years than in the earlier survey years, consistent with trends in other African countries [9]. Although we assume that levels of antenatal bias are constant over time, we make implicit allowance for the possibility of error due to non-constant bias by including the ‘model error’ term defined previously.

Table S1: Differences (on logit scale) between antenatal survey and household survey estimates of HIV prevalence

|  | 2005 | 2008 | 2012 | 2017 | Average^*^ |
| --- | --- | --- | --- | --- | --- |
| Eastern Cape (EC) | 0.825 | 0.755 | 0.502 | 0.389 | 0.618 |
| Free State (FS) | 0.604 | 0.770 | 0.608 | 0.309 | 0.573 |
| Gauteng (GT) | 0.938 | 0.867 | 0.678 | 0.634 | 0.779 |
| KwaZulu-Natal (KZ) | 0.828 | 0.596 | 0.434 | 0.526 | 0.596 |
| Limpopo (LP) | 0.796 | 0.497 | 0.575 | 0.335 | 0.551 |
| Mpumalanga (MP) | 0.575 | 0.606 | 0.685 | 0.529 | 0.599 |
| Northern Cape (NC) | 0.831 | 0.670 | 0.472 | 0.217 | 0.547 |
| North West (NW) | 0.753 | 0.737 | 0.506 | 0.190 | 0.546 |
| Western Cape (WC) | 1.729 | 1.232 | 0.877 | 0.420 | 1.065 |
| Average | 0.875 | 0.748 | 0.593 | 0.394 | 0.653 |

* The average serves as the mean of the prior distribution for the *b_i_* parameter.

The $\sigma_{i,t}^{2}$ parameters have been estimated from the published 95% confidence intervals around the antenatal survey estimates, in 1998 and subsequent years. Prior to 1998, the published 95% confidence intervals were calculated on the assumption of simple random sampling (SRS), i.e. not reflecting the clustering associated with the sampling of antenatal clinics. As these confidence intervals would have exaggerated the precision associated with the prevalence estimates, we recalculated the standard errors by inflating the published standard errors up to 1998 by province-specific adjustment factors. The adjustment factor was calculated as the average ratio of the published standard error to the SRS standard error over the 2003-2005 period (the only period for which we had sufficient data to calculate both standard error estimates in all provinces). The $\sigma_{i,t}^{2}$ parameters up to 1998 were then estimated from these inflated standard error estimates. Confidence intervals have not been reported for the 2014 and 2015 survey estimates, and the standard errors in these two years have therefore been assumed to be the same as in 2013.

Having obtained the $\sigma_{i,t}^{2}$ parameters, the variance of the model error ($\sigma_{i}^{2}$) is estimated using the formula

$\hat{\sigma}_{i}^{2}=\frac{1}{23}\sum_{t} \left(\log\left(\frac{y_{i,t}}{1-y_{i,t}}\right)-\log\left(\frac{H_{i,t}\left(\mathbf{\varphi}\right)}{1-H_{i,t}\left(\mathbf{\varphi}\right)}\right)-b_{i}\right)^{2}-\sigma_{i,t}^{2}$.

The likelihood in respect of the antenatal data is then calculated based on the assumption that the error terms are normally distributed:

,

where $\mathbf{y}_{i}$ represents the vector of *y_i_*,*_t_* values, across calendar years 1994 to 2015 and 2017.

## **2.2 Method 2: Adjusted definition of antenatal bias**

In contrast to the previously-described approach, this approach involves calibrating the model to *age-specific* HIV prevalence data. Suppose that $H_{i,x,t}\left(\mathbf{\varphi}\right)$ is the model estimate of HIV prevalence in pregnant women in province *i*, in age group *x* and year *t*, where the vector $\mathbf{\varphi}$ represents the values of the model input parameters. The corresponding prevalence of HIV actually measured in the antenatal survey is represented by $y_{i,x,t}$. It is assumed that if $\mathbf{\varphi}$ is the true set of parameter values, then the difference between the logit-transformed model estimate and the logit-transformed observed prevalence is normally distributed. The mean of this normal distribution again represents the extent of antenatal bias, although here the antenatal bias arises due to different factors, such as the exclusion of women receiving private antenatal care from the antenatal survey. The variance of the distribution is again assumed to be composed of a ‘survey error’ term and a ‘model error’ term. More formally, it is assumed that

$\log\left(\frac{y_{i,x,t}}{1-y_{i,x,t}}\right)=\log\left(\frac{H_{i,x,t}\left(\mathbf{\varphi}\right)}{1-H_{i,x,t}\left(\mathbf{\varphi}\right)}\right)+b_{i}+m_{i,x,t}+\varepsilon_{i,x,t}$,

where $b_{i}$ is the antenatal bias parameter, $m_{i,x,t}\sim N\left(0,\sigma_{i}^{2}\right)$ and $\varepsilon_{i,x,t}\sim N\left(0,\sigma_{i,x,t}^{2}\right)$.

In the calibration of the national model to age-specific antenatal data, the antenatal bias was a free parameter that was estimated to be 0.426 [10]. To adopt the same approach as in the national model, of treating the antenatal bias as a free parameter, would lead to too much variation in the extent of the antenatal bias between provinces, and initial attempts to apply this approach led to implausible HIV estimates in some provinces. Our approach is therefore to constrain the extent of the antenatal bias by specifying prior distributions on the antenatal bias parameters, similar to the approach adopted in other recent modelling studies [11, 12]. The mean of the prior distribution is assumed to differ between provinces, depending on the fraction of the population that uses private healthcare facilities, since much of the antenatal bias is attributable to the exclusion of pregnant women attending private facilities from the antenatal survey. Mathematically, the prior mean is estimated by assuming that

$\bar{b}_{i}=\psi+\chi\omega_{i}$ ,

where $\bar{b}_{i}$ is the mean of the prior distribution on *b_i_*, *ψ* is the antenatal bias that would be expected if all pregnant women used public health facilities, *χ* is the difference in HIV prevalence between women using private antenatal services and women using public antenatal services (on the logit scale), and $\omega_{i}$ is the proportion of pregnant women in province *i* who use private antenatal services. Estimates of differences in HIV prevalence between pregnant women using private and public antenatal services are very limited, but a 1998 survey in KwaZulu-Natal found a prevalence of 14.0% in pregnant women using private facilities [13], which compared with a prevalence of 32.5% in public antenatal clinics in KwaZulu-Natal in the same year [14]. This suggests a value of *χ* equal to 1.08. Estimates of the $\omega_{i}$ values are not directly reported, but we have approximated these values using the proportions of adults who reported being members of medical schemes in the 2003 DHS [15], as shown in Table S2. Given that this proportion is 14.2% for the country as a whole, and given the previously-estimated antenatal bias of 0.426 for the country as a whole, we estimate the *ψ* parameter as 0.272 (0.426 – 1.08 × 0.142). These parameter values are entered into equation (2) to obtain the prior means for each province, as shown in Table S2. As might be expected, the estimated antenatal bias levels are greatest in Gauteng and the Western Cape, the two provinces with the highest levels of medical scheme membership.

Table S2: Antenatal bias levels by province

| Province | EC | FS | GT | KZ | LM | MP | NC | NW | WC |
| --- | --- | --- | --- | --- | --- | --- | --- | --- | --- |
| % in medical  schemes ($\omega_{i}$) | 10.3% | 11.0% | 22.0% | 9.6% | 8.4% | 12.5% | 15.8% | 9.4% | 20.3% |
| Antenatal  bias ($\bar{b}_{i}$) | 0.383 | 0.391 | 0.510 | 0.376 | 0.363 | 0.407 | 0.443 | 0.374 | 0.492 |

For provincial abbreviations, see Table S1.

To represent the uncertainty around the antenatal bias parameters, we again use gamma prior distributions. The coefficient of variation around the antenatal bias parameter estimated for the country as a whole is 3.9%, but because factors other than private sector bias might account for differences in antenatal bias across provinces, it is appropriate to assume a larger coefficient of variation to represent the uncertainty around the antenatal bias at a provincial level. We therefore assume a coefficient of variation of 15% for all provinces when assigning prior distributions to represent the uncertainty around $\bar{b}_{i}$.

The $\sigma_{i,x,t}^{2}$ parameters have again been estimated from the published 95% confidence intervals around the antenatal survey estimates, as described previously. Having obtained the $\sigma_{i,x,t}^{2}$ parameters, the variance of the model error ($\sigma_{i}^{2}$) is estimated using the formula

$\hat{\sigma}_{i}^{2}=\frac{1}{115}\sum_{x} \sum_{t} \left(\log\left(\frac{y_{i,x,t}}{1-y_{i,x,t}}\right)-\log\left(\frac{H_{i,x,t}\left(\mathbf{\varphi}\right)}{1-H_{i,x,t}\left(\mathbf{\varphi}\right)}\right)-b_{i}\right)^{2}-\sigma_{i,x,t}^{2}$.

The likelihood in respect of the antenatal data is then calculated based on the assumption that the error terms are normally distributed:

,

where $\mathbf{y}_{i}$ represents the vector of $y_{i,x,t}$ values, across calendar years 1994 to 2015 and 2017.

## **2.3 Method 3: Recency-adjusted definition of antenatal bias**

The approach to specifying antenatal bias is similar to that in the previous section:

$\log\left(\frac{y_{i,x,t}}{1-y_{i,x,t}}\right)=\log\left(\frac{H_{i,x,t}\left(\mathbf{\varphi}\right)\lambda_{i,x,t}\left(\mathbf{\varphi}\right)^{\theta}}{1-H_{i,x,t}\left(\mathbf{\varphi}\right)\lambda_{i,x,t}\left(\mathbf{\varphi}\right)^{\theta}}\right)+b_{i}+m_{i,x,t}+\varepsilon_{i,x,t}$,

where *λ_i_*,*_x_*,*_t_*(**φ**) is the fraction of HIV-positive women aged *x* to *x*+4 who were infected in the last 12 months, as at time *t*, *θ* is a scaling factor, and all other terms are defined in the same way as before. We set *θ* = 0.04, as described in the main text, because this appears to minimize the variance of the model errors associated with the constant antenatal bias assumption.

For the purpose of setting the prior on *b_i_*, we note that

$$\log\left(\frac{H_{i,x,t}\left(\mathbf{\varphi}\right)\lambda_{i,x,t}\left(\mathbf{\varphi}\right)^{\theta}}{1-H_{i,x,t}\left(\mathbf{\varphi}\right)\lambda_{i,x,t}\left(\mathbf{\varphi}\right)^{\theta}}\right)=\log\left(\frac{H_{i,x,t}\left(\mathbf{\varphi}\right)}{1-H_{i,x,t}\left(\mathbf{\varphi}\right)\lambda_{i,x,t}\left(\mathbf{\varphi}\right)^{\theta}}\right)+\theta\log\left(\lambda_{i,x,t}\left(\mathbf{\varphi}\right)\right)$$

$$\approx\log\left(\frac{H_{i,x,t}\left(\mathbf{\varphi}\right)}{1-H_{i,x,t}\left(\mathbf{\varphi}\right)}\right)+\theta\log\left(\lambda_{i,x,t}\left(\mathbf{\varphi}\right)\right)$$

if *θ* is close to zero. This means that

$$\log\left(\frac{y_{i,x,t}}{1-y_{i,x,t}}\right)\approx\log\left(\frac{H_{i,x,t}\left(\mathbf{\varphi}\right)}{1-H_{i,x,t}\left(\mathbf{\varphi}\right)}\right)+\theta\log\left(\lambda_{i,x,t}\left(\mathbf{\varphi}\right)\right)+b_{i}+m_{i,x,t}+\varepsilon_{i,x,t}$$

which is the same as the equation under the adjusted approach (described in the previous section), except for the inclusion of the *θ* × log(*λ_i_*,*_x_*,*_t_*(**φ**)) term. This means that the prior on the antenatal bias parameter, under the recency-adjusted approach, should be higher than that under the adjusted approach by -*θ* × log(*λ_i_*,*_x_*,*_t_*(**φ**)). In our simulations, *λ_i_*,*_x_*,*_t_*(**φ**) is usually in the range of 0.02 to 0.50 (see Figure 5(d) in the main text), and thus the -*θ* × log(*λ_i_*,*_x_*,*_t_*(**φ**)) term is usually in the range of 0.03 to 0.16. We take the midpoint of this range (0.10) as the addition to the prior means specified in the previous section for the purpose of getting the means on the prior distributions under the recency-adjusted approach. The means of the gamma prior distributions are shown in Table S3, and the standard deviations of the prior distributions are calculated as 15% of the prior means.

Table S3: Antenatal bias levels by province (means of prior distributions)

| Province | EC | FS | GT | KZ | LM | MP | NC | NW | WC |
| --- | --- | --- | --- | --- | --- | --- | --- | --- | --- |
| Adjusted antenatal  bias | 0.383 | 0.391 | 0.510 | 0.376 | 0.363 | 0.407 | 0.443 | 0.374 | 0.492 |
| Recency-adjusted  antenatal bias | 0.483 | 0.491 | 0.610 | 0.476 | 0.463 | 0.507 | 0.543 | 0.474 | 0.592 |

For provincial abbreviations, see Table 1.

The $\sigma_{i,x,t}^{2}$ parameters are estimated in the same way as described previously. The variance of the model error ($\sigma_{i}^{2}$) is estimated using the formula

$\hat{\sigma}_{i}^{2}=\frac{1}{115}\sum_{x} \sum_{t} \left(\log\left(\frac{y_{i,x,t}}{1-y_{i,x,t}}\right)-\log\left(\frac{H_{i,x,t}\left(\mathbf{\varphi}\right)\lambda_{i,x,t}\left(\mathbf{\varphi}\right)}{1-H_{i,x,t}\left(\mathbf{\varphi}\right)\lambda_{i,x,t}\left(\mathbf{\varphi}\right)}\right)-b_{i}\right)^{2}-\sigma_{i,x,t}^{2}$.

The likelihood in respect of the antenatal data is then calculated as:

.

## **2.4 Results of calibration**

Figure S3 shows the results of the calibration to the antenatal and household survey data, obtained using the unadjusted definition of antenatal bias. Although the model fits to the data generally appear acceptable, the fit to the antenatal survey data is poor in Gauteng province.

Figure S3: Calibration based on unadjusted definition of antenatal bias

Figures S4-S12 show the calibration to the age-specific antenatal survey data, for both the adjusted and recency-adjusted approaches. In most cases, the model fits to the data are very similar when using the adjusted and recency-adjusted approaches, although the model estimates a slightly lower HIV prevalence in older pregnant women when using the recency-adjusted approach. In most provinces, the model does not fit HIV prevalence in the 20-24 age group well.

Figure S4: HIV prevalence in pregnant women in the Eastern Cape

Figure S5: HIV prevalence in pregnant women in the Free State

Figure S6: HIV prevalence in pregnant women in Gauteng

Figure S7: HIV prevalence in pregnant women in KwaZulu-Natal

Figure S8: HIV prevalence in pregnant women in Limpopo

Figure S9: HIV prevalence in pregnant women in Mpumalanga

Figure S10: HIV prevalence in pregnant women in Northern Cape

Figure S11: HIV prevalence in pregnant women in North West

Figure S12: HIV prevalence in pregnant women in Western Cape

Table S4 compares the estimates of HIV incidence obtained using the unadjusted approach to defining antenatal bias with those obtained using the adjusted approach. Although HIV incidence rates in 1997 are consistently higher when estimated using the unadjusted approach to defining antenatal bias than when estimated using the adjusted approach, the same is not true of HIV incidence rates in 2007 or 2017, and there is also no consistent difference when comparing the age-disaggregated HIV incidence estimates in 2017 (for women). What is most remarkable is that the coefficients of variation (for all HIV incidence estimates) are consistently higher when using the unadjusted approach than when using the adjusted approach, i.e. there is a substantial improvement in precision around HIV incidence estimates when using age-specific HIV prevalence data in the model calibration. The average reduction in the coefficient of variation is more substantial in 2017 (33% reduction) than in 2007 (29% reduction) and in 1997 (26% reduction), and is also more substantial when considering age-specific HIV incidence rates in 2017: the average reduction in coefficients of variation is 39% when considering females aged 15-24 and 49% when considering women aged 25-49. Thus the gains in precision from using age-specific data in the model calibration are most significant when estimating HIV incidence in recent years and when estimating HIV incidence in more narrowly-defined age groups.

Table S4: HIV incidence estimates

|  | EC | FS | GT | KZ | LP | MP | NC | NW | WC |
| --- | --- | --- | --- | --- | --- | --- | --- | --- | --- |
| HIV incidence in 15-49 year olds in 2017 | | | | | | | | | |
| Unadjusted | 1.12% | 0.94% | 0.91% | 1.02% | 0.64% | 1.16% | 0.48% | 1.21% | 0.42% |
| CoV | 8.99% | 11.03% | 9.69% | 10.24% | 10.04% | 9.04% | 13.49% | 8.76% | 15.32% |
| Adjusted | 1.07% | 0.96% | 0.80% | 1.13% | 0.52% | 1.06% | 0.40% | 1.11% | 0.51% |
| CoV | 5.00% | 6.15% | 6.52% | 6.68% | 7.01% | 7.46% | 8.63% | 6.38% | 10.45% |
| HIV incidence in 15-49 year olds in 2007 | | | | | | | | | |
| Unadjusted | 2.04% | 2.10% | 1.54% | 2.62% | 1.52% | 2.42% | 1.05% | 1.82% | 0.76% |
| CoV | 4.57% | 5.13% | 5.70% | 3.76% | 4.77% | 4.67% | 6.16% | 4.90% | 7.80% |
| Adjusted | 2.00% | 2.04% | 1.52% | 2.73% | 1.41% | 2.29% | 0.98% | 1.72% | 0.82% |
| CoV | 3.09% | 3.84% | 4.33% | 2.78% | 3.12% | 3.20% | 4.04% | 3.86% | 5.02% |
| HIV incidence in 15-49 year olds in 1997 | | | | | | | | | |
| Unadjusted | 2.68% | 3.26% | 2.42% | 3.98% | 1.94% | 3.48% | 1.46% | 3.11% | 0.73% |
| CoV | 4.86% | 5.29% | 5.47% | 4.18% | 5.49% | 4.39% | 6.36% | 4.77% | 9.45% |
| Adjusted | 2.04% | 2.86% | 2.24% | 3.63% | 1.44% | 3.07% | 1.24% | 2.84% | 0.65% |
| CoV | 3.66% | 4.02% | 4.34% | 3.57% | 3.85% | 2.94% | 5.08% | 3.52% | 5.27% |
| HIV incidence in 15-24 year old women in 2017 | | | | | | | | | |
| Unadjusted | 2.17% | 1.48% | 1.66% | 2.16% | 1.06% | 2.44% | 0.92% | 2.00% | 0.85% |
| CoV | 11.22% | 13.50% | 10.87% | 11.42% | 14.32% | 10.38% | 14.56% | 11.43% | 15.57% |
| Adjusted | 2.11% | 1.87% | 1.36% | 2.26% | 0.97% | 2.19% | 0.76% | 2.07% | 0.96% |
| CoV | 5.10% | 6.09% | 7.71% | 7.51% | 6.88% | 7.77% | 9.95% | 7.36% | 10.21% |
| HIV incidence in 25-49 year old women in 2017 | | | | | | | | | |
| Unadjusted | 0.95% | 1.17% | 0.89% | 0.81% | 0.76% | 0.90% | 0.43% | 1.36% | 0.36% |
| CoV | 15.14% | 15.79% | 14.50% | 10.98% | 14.34% | 17.20% | 21.78% | 14.37% | 22.46% |
| Adjusted | 0.94% | 0.98% | 0.87% | 1.04% | 0.57% | 0.91% | 0.37% | 1.09% | 0.48% |
| CoV | 6.31% | 7.66% | 7.77% | 5.99% | 8.56% | 9.30% | 9.70% | 7.44% | 11.39% |

For provincial abbreviations, see Table S1. The coefficient of variation (CoV) is calculated as the standard deviation of the posterior distribution divided by the posterior mean.

Figure S13 compares a series of model outputs, with the results obtained using the adjusted definition on the x axis, and the results obtained using the recency-adjusted definition on the y axis (the dashed line in each panel represents the line on which all points would lie if there was exact consistency between the two calibration procedures). The use of the recency adjustment leads to slightly lower estimates of HIV prevalence in 1997 but slightly higher estimates of HIV prevalence in 2017 (panels a and b). HIV incidence are very similar in 1997, but the recency-adjusted estimates of HIV incidence in 2017 are slightly higher than those obtained using the adjusted definition of antenatal bias (panels c and d). As estimates of the fraction of HIV-positive individuals who are diagnosed tend to decrease as HIV incidence increases, the recency-adjusted estimates of the levels of HIV diagnosis are lower than those obtained with the adjusted definition of antenatal bias (panel e). Estimates of ART coverage are also lower when using the recency-adjusted definition of antenatal bias (panel f). Although the recency adjustment leads to higher HIV incidence in 2017, the difference is much more marked in the 25-49 age group than in the 15-24 age group (panels g and h), with the result that adolescent girls and young women (aged 15-24) account for a lower proportion of new infections in the 15-49 age group, when using the recency adjustment (panel i).

Figure S13: Comparison of the results of the Thembisa model when calibrated using the adjusted definition of antenatal bias (x axis) against those obtained using the recency-adjusted definition of antenatal bias (y axis)

Blue dots represent the results for each of the nine provinces of South Africa (for provincial abbreviations, see Table S1). The dashed line represents the line of equality between the two calibration procedures.

## **2.5 Sensitivity analysis: Calibration without antenatal HIV prevalence data**

As countries in sub-Saharan Africa have relied increasingly on regular household surveys to estimate trends in HIV prevalence and incidence, one might question whether it is still important to calibrate mathematical models to antenatal survey data. Indeed, several recent models of HIV epidemics in sub-Saharan Africa have been calibrated only to household survey data [16-18]. If antenatal survey HIV prevalence data do not influence recent model estimates of HIV prevalence and HIV incidence, then the question of bias in antenatal survey data becomes less important.

We therefore assess the extent to which model estimates of HIV incidence and prevalence change when the Thembisa model is not calibrated to antenatal survey data (i.e. only the HIV prevalence data from the household surveys in 2005, 2008, 2012, 2016 and 2017 are used in calibration). Figure S14 compares the calibration to the household survey data when including the antenatal HIV prevalence data (using the recency-adjusted definition of antenatal bias) and the calibration when excluding the antenatal survey data. The model fits to the HIV prevalence data from the household surveys are similarly good in most provinces, although in the Western Cape the exclusion of the antenatal survey data allows a better fit to the household survey data. Estimates of HIV prevalence in 15-49 year olds in recent years appear relatively insensitive to the exclusion of the antenatal survey data in the calibration, but in the earlier stages of the HIV epidemic, estimates of HIV prevalence are substantially different depending on whether the antenatal HIV data are included, in some provinces.

Figure S14: HIV prevalence in adults aged 15-49

Figure S15 compares the model estimates of adult HIV incidence when including only the household survey HIV prevalence data in the calibration, and when including both the antenatal and household survey HIV prevalence data. In most provinces, the trend in HIV incidence over the most recent decade is similar regardless of whether the antenatal survey data are included, but in the Western Cape the exclusion of the antenatal survey data leads to a later HIV incidence peak and hence a less substantial decline in HIV incidence. In most provinces (most noticeably Eastern Cape, Free State, Limpopo, Mpumalanga and Western Cape), the estimates of HIV incidence trends in the early stages of the HIV epidemic (before 2000) are also sensitive to the exclusion of the antenatal survey data in the calibration. Except in Eastern Cape and KwaZulu-Natal, estimates of HIV incidence are higher in the most recent years when excluding the antenatal survey data from the calibration. As shown in Figure 8b of the main text, the exclusion of the antenatal survey data from the model calibration also leads to substantially wider 95% confidence intervals around the HIV incidence estimates.

Figure S15: HIV incidence in adults aged 15-49

## **2.6 Sensitivity analysis: Alternative transformations**

A number of possible transformations can be used when defining antenatal bias. Figure S16a compares the effect of using different transformations when defining the unadjusted measure of antenatal bias, in the hypothetical scenario in which HIV prevalence in pregnant women is consistently 1.2 times the HIV prevalence in the general population. The logit transformation is similar to the probit transformation: both tend to inflate differences in HIV prevalence as prevalence increases (i.e. the bias appears greater at higher HIV prevalence levels). The log transformation, however, maintains a constant antenatal bias. Figure S16b shows the effect that the assumption of constant bias has on the ratio of HIV prevalence in the general population to that in pregnant women, when the antenatal bias is fixed at 0.2. The log transformation yields a constant ratio, while the other two adjustments lead to increasing ratios as HIV prevalence increases.

Figure S16: Effect of different transformations when assuming either (a) a constant ratio of antenatal prevalence to general population prevalence (1.2), or (b) a constant antenatal bias (0.2)

To assess the effect of using different transformations, we refit the Thembisa model using the unadjusted antenatal bias definition, replacing the default logit transformation in Thembisa with log and probit transformation. We perform this comparison for two provinces, Western Cape and KwaZulu-Natal, as they lie at opposite ends of the HIV prevalence spectrum. The approach to calibrating the model using the logit transformation has already been explained in section 2.1. For the purpose of applying the log and probit transformations, we follow the same approach, but using different priors for the antenatal bias parameter. As in section 2.1, we calculate the prior means by calculating the average difference in HIV prevalence between antenatal surveys and general populations surveys, using the respective transformations (Table S5), and we calculate the standard deviation of the prior distribution using an assumed coefficient of variation of 0.15.

Table S5: Derivation of prior means for antenatal bias, using alternative transformations

|  | 2005 | 2008 | 2012 | 2017 | Average^*^ |
| --- | --- | --- | --- | --- | --- |
| Western Cape |  |  |  |  |  |
| Antenatal HIV prevalence | 15.7% | 16.1% | 16.9% | 18.0% |  |
| Household survey prevalence | 3.2% | 5.3% | 7.8% | 12.6% |  |
| Logit difference | 1.729 | 1.232 | 0.877 | 0.420 | 1.065 |
| Log difference | 1.591 | 1.111 | 0.773 | 0.357 | 0.958 |
| Probit difference | 0.845 | 0.626 | 0.461 | 0.230 | 0.541 |
| KwaZulu-Natal |  |  |  |  |  |
| Antenatal HIV prevalence | 39.1% | 38.7% | 37.4% | 38.5% |  |
| Household survey prevalence | 21.9% | 25.8% | 27.9% | 27.0% |  |
| Logit difference | 0.828 | 0.596 | 0.434 | 0.526 | 0.596 |
| Log difference | 0.580 | 0.405 | 0.293 | 0.355 | 0.408 |
| Probit difference | 0.499 | 0.362 | 0.265 | 0.320 | 0.362 |

* The average serves as the mean of the prior distribution for the *b_i_* parameter.

Figure S17 shows the resulting model estimates of HIV prevalence and incidence trends for each of the three transformations. Overall, the choice of the transformation has little influence on the model estimates of HIV incidence and prevalence, especially in the period after 2005. In the earlier stages of the HIV epidemic, the probit transformation leads to slightly lower estimates of HIV incidence and prevalence, while the log transformation leads to slightly higher estimates of prevalence and incidence, and the logit transformation produces estimates that lie between the probit and log transformations. These differences can be explained in terms of the patterns shown in Figure S16b: the assumption of a constant antenatal bias on the probit scale amplifies the relative differences in prevalence to a greater extent than the other transformations, and thus for a given observed antenatal prevalence, the probit transformation will tend to suggest a lower HIV prevalence in the general population early in the epidemic, relative to the other transformations.

Figure S17: Effect of different transformations on estimates of HIV prevalence and incidence in the Thembisa model

# **3. Additional MicroCOSM outputs**

Figure S18, which is comparable with Figure 4 in the main text, shows the effect of changing the assumptions about changes in hormonal contraceptive use over time. In the base scenario, there is assumed to be an 80% reduction in the rate of hormonal contraceptive uptake among young women over the period between 1997 and 2005. In the counterfactual scenario, we assess what the trend in antenatal bias would have been if there had been no change in rates of hormonal contraceptive uptake over time. Overall, there is little change in antenatal bias in the counterfactual scenario, although antenatal bias increases slightly in the 15-24 age group.

Figure S18: Effect of changes in hormonal contraceptive use in young women on antenatal bias

Antenatal bias is calculated using the adjusted definition (on a probit scale). In both panels, the blue line corresponds to the baseline scenario. Solid lines represent means from 100 simulations.

# **References**

1. Johnson LF, Dorrington RE. Modelling the impact of HIV in South Africa's provinces: 2019 update. University of Cape Town; 2019. Available: <https://www.thembisa.org/>

2. Raftery AE, Bao L. Estimating and projecting trends in HIV/AIDS generalized epidemics using Incremental Mixture Importance Sampling. *Biometrics* 2010; **66**:1162-1173.

3. Shisana O, Rehle T, Simbayi LC, Parker W, Zuma K, Bhana A*, et al.* South African National HIV Prevalence, HIV Incidence, Behaviours and Communication Survey, 2005. Cape Town: HSRC Press; 2005. Available: <http://www.hsrcpress.ac.za>. Accessed 1 Dec 2005

4. Shisana O, Rehle T, Simbayi LC, Zuma K, Jooste S, Pillay-van Wyk V*, et al.* South African national HIV prevalence, incidence, behaviour and communication survey, 2008: A turning tide among teenagers? Cape Town: Human Sciences Research Council; 2009. Available: <http://www.hsrcpress.ac.za>. Accessed 9 June 2009

5. Shisana O, Rehle T, Simbayi LC, Zuma K, Jooste S, Zungu N*, et al.* South African National HIV Prevalence, Incidence, and Behaviour Survey, 2012. Cape Town: Human Sciences Research Council; 2014. Available: <http://www.hsrc.ac.za/en/research-outputs/view/6871>. Accessed 16 April 2014

6. Department of Health, Statistics South Africa, South African Medical Research Council, ICF. South Africa Demographic and Health Survey 2016. Pretoria; 2019. Available: <https://www.dhsprogram.com/pubs/pdf/FR337/FR337.pdf>. Accessed 19 March 2019

7. Human Sciences Research Council. HIV Impact Assessment Summary. 2018. Available: <http://serve.mg.co.za/content/documents/2018/07/17/7M1RBtUShKFJbN3NL1Wr_HSRC_HIV_Survey_Summary_2018.pdf>. Accessed 18 July 2018

8. Woldesenbet S, Kufa T, Lombard C, Manda S, Ayalew K, Cheyip M*, et al.* The 2017 National Antenatal Sentinel HIV Survey, South Africa. National Department of Health; 2019. Available: <http://www.nicd.ac.za/wp-content/uploads/2019/07/Antenatal_survey-report_24July19.pdf>. Accessed 29 Nov 2019

9. Eaton JW, Rehle TM, Jooste S, Nkambule R, Kim AA, Mahy M*, et al.* Recent HIV prevalence trends among pregnant women and all women in sub-Saharan Africa: implications for HIV estimates. *AIDS* 2014; **28 (Suppl 4)**:S507-514.

10. Johnson L. THEMBISA version 1.0: A model for evaluating the impact of HIV/AIDS in South Africa. Centre for Infectious Disease Epidemiology and Research, University of Cape Town; 2014. Available: <http://www.thembisa.org/content/downloadPage/WPversion1>. Accessed 21 April 2016

11. Brown T, Bao L, Eaton JW, Hogan DR, Mahy M, Marsh K*, et al.* Improvements in prevalence trend fitting and incidence estimation in EPP 2013. *AIDS* 2014; **28 (Suppl 4)**:S415-425.

12. Eaton JW, Hallett TB. Why the proportion of transmission during early-stage HIV infection does not predict the long-term impact of treatment on HIV incidence. *Proc Natl Acad Sci U S A* 2014; **111**:16202-16207.

13. Wilkinson D. HIV infection among pregnant women in the South African private medical sector. *AIDS* 1999; **13**:1783.

14. Department of Health. 1998 national HIV sero-prevalence survey of women attending public antenatal clinics in South Africa. 1999.

15. Department of Health. South Africa Demographic and Health Survey 2003: Preliminary Report. Pretoria; 2004. Available: <http://www.doh.gov.za/docs/reports/2003/sadhs2003/part2.pdf>. Accessed 6 Jan 2012

16. Phillips A, Cambiano V, Johnson L, Nakagawa F, Homan R, Meyer-Rath G*, et al.* Potential impact and cost-effectiveness of condomless-sex-concentrated PrEP in KwaZulu-Natal accounting for drug resistance. *J Infect Dis* 2020; [In press].

17. Akullian A, Morrison M, Garnett GP, Mnisi Z, Lukhele N, Bridenbecker D*, et al.* The effect of 90-90-90 on HIV-1 incidence and mortality in eSwatini: a mathematical modelling study. *Lancet HIV* 2020; **7**:e348-e358.

18. Cremin I, McKinnon L, Kimani J, Cherutich P, Gakii G, Muriuki F*, et al.* PrEP for key populations in combination HIV prevention in Nairobi: a mathematical modelling study. *Lancet HIV* 2017; **4**:e214-e222.
